# Human Skeletal Muscle Possesses an Epigenetic Memory of Hypertrophy

**DOI:** 10.1038/s41598-018-20287-3

**Published:** 2018-01-30

**Authors:** Robert A. Seaborne, Juliette Strauss, Matthew Cocks, Sam Shepherd, Thomas D. O’Brien, Ken A. van Someren, Phillip G. Bell, Christopher Murgatroyd, James P. Morton, Claire E. Stewart, Adam P. Sharples

**Affiliations:** 1 Institute for Science and Technology in Medicine (ISTM), School of Medicine, Keele University, Staffordshire, United Kingdom; 2 Research Institute for Sport and Exercise Sciences, Liverpool John Moores University, Liverpool, United Kingdom; 3 Department of Sport, Exercise and Rehabilitation, Northumbria University, Newcastle upon Tyne, United Kingdom; 4 School of Healthcare Science, Manchester Metropolitan University, Manchester, United Kingdom

## Abstract

It is unknown if adult human skeletal muscle has an epigenetic memory of earlier encounters with growth. We report, for the first time in humans, genome-wide DNA methylation (850,000 CpGs) and gene expression analysis after muscle hypertrophy (loading), return of muscle mass to baseline (unloading), followed by later hypertrophy (reloading). We discovered increased frequency of hypomethylation across the genome after reloading (18,816 CpGs) versus earlier loading (9,153 CpG sites). We also identified AXIN1, GRIK2, CAMK4, TRAF1 as hypomethylated genes with enhanced expression after loading that maintained their hypomethylated status even during unloading where muscle mass returned to control levels, indicating a memory of these genes methylation signatures following earlier hypertrophy. Further, UBR5, RPL35a, HEG1, PLA2G16, SETD3 displayed hypomethylation and enhanced gene expression following loading, and demonstrated the largest increases in hypomethylation, gene expression and muscle mass after later reloading, indicating an epigenetic memory in these genes. Finally, genes; GRIK2, TRAF1, BICC1, STAG1 were epigenetically sensitive to acute exercise demonstrating hypomethylation after a single bout of resistance exercise that was maintained 22 weeks later with the largest increase in gene expression and muscle mass after reloading. Overall, we identify an important epigenetic role for a number of largely unstudied genes in muscle hypertrophy/memory.

## Introduction

Numerous studies demonstrate that skeletal muscle can be programed, where early life exposure to environmental stimuli lead to a sustained alteration of skeletal muscle phenotype in later life [reviewed in ref.^1^]. This has been demonstrated in mammalian models in which reduced nutrient availability during gestation impairs skeletal muscle fibre number, composition (fast/slow fibre proportions) and size of the offspring^1^. Epidemiological studies in human ageing cohorts also suggest that low birth weight and gestational malnutrition are strongly associated with reduced skeletal muscle size, strength and gait speed in older age^2,3^. Driven by encounters with the environment, foetal programming in skeletal muscle has been attributed in part to epigenetics^4,5^, which refers to alterations in gene expression as a result of non-genetic structural modifications of DNA and/or histones^6^. Despite these compelling data, it is unknown if adult skeletal muscle possesses the capacity to respond differently to environmental stimuli in an adaptive or maladaptive manner if the stimuli have been encountered previously, a concept recently defined as skeletal muscle memory^1^, or if this process is epigenetically regulated. Indeed, it is known that skeletal muscle cells retain information or ‘remember’ the stem cell niche of the donor once derived *in-vitro* from physically active^7^ obese^8,9^ and sarcopenic individuals [recently reviewed in ref.^1^]. Our group were the first to demonstrate this phenomenon, where human muscle stem cells derived from the skeletal muscle of cancer patients exhibited overactive proliferation versus age matched control cells^10^. These studies collectively suggest that skeletal muscle cells could be epigenetically regulated, as they appear to not only retain information from the environmental niche from which they originated, but also to pass this molecular ‘signature’ onto future daughter cell progeny *in-vitro*. Furthermore, we have recently reported that mouse skeletal muscle cells (C2C12), following an early-life inflammatory stress, pass molecular information onto future generations (30 cellular divisions), through a process of DNA methylation^11^. Importantly, the cells that encountered catabolic inflammatory stress in their earlier proliferative life had impaired differentiation capacity when encountering the same inflammatory stress in later proliferative life^11^. It has therefore been proposed that a memory and susceptibility of skeletal muscle to repeated encounters with inflammation may be controlled by epigenetic modifications such as DNA methylation, a phenomenon we have termed skeletal muscle ‘epi-memory’^1^.

Mouse skeletal muscle *in-vivo* also appears to possess a memory from the anabolic growth steroid sex hormone, testosterone. Where testosterone induced hypertrophy over a period of 3 months, resulted in enhanced incorporation of myonuclei within muscle fibres^12,13^. These myonuclei were retained even following testosterone withdrawal and the return of muscle mass to baseline^12,13^. Most notably the mice exposed to earlier life testosterone, exhibited a 31% increase in muscle cross-sectional area following mechanical loading versus control mice that failed to grow in the same period of time^12,13^. This suggests an enhanced response to load induced muscle hypertrophy when earlier life growth from testosterone had been encountered, and therefore corresponds with the previously highlighted definition of muscle memory by Sharples *et al*.^1^. However, epigenetics has not been studied in this model, and specifically genome-wide DNA methylation has not been investigated after adult human skeletal muscle growth (hypertrophy) alone, or in skeletal muscle that has experienced later growth, to investigate if skeletal muscle possesses an epigenetic memory from earlier life encounters with hypertrophy.

To provide parallel insights into the effect of the environment on genome-wide methylation changes in skeletal muscle, recent studies have suggested that even an acute period of increased fat intake can alter the human DNA methylome of CpGs in over 6,500 genes^14^. Like previous studies demonstrating rapid and dynamic alterations in DNA methylation in skeletal muscle tissue after acute metabolic stress (aerobic exercise)^15,16^ or disuse atrophy in rats^17^, this study also suggested that large scale epigenetic modifications can occur very rapidly in skeletal muscle, after only 5 days of high fat feeding. However, the authors also demonstrated a maintenance of methylation following cessation of the high fat diet^14^. Where after 8 weeks of returning to a normal diet, not all of the altered methylation, particularly hypermethylation, was fully returned to baseline control levels^14^. This therefore suggests that in response to an acute negative environmental stress, DNA methylation could be retained and accumulated over time. Indeed, human skeletal muscle cells isolated from aged donors demonstrated a genome wide hypermethylated profile versus young adult tissue^18^. Therefore, because DNA methylation, particularly within promoter or enhancer regions of genes, generally leads to suppressed gene expression^19^, accumulation of high DNA methylation (hypermethylation) following a high fat diet and/or ageing could lead to universally suppressed gene expression. It may therefore be hypothesised that positive environmental encounters, such as muscle growth stimuli, may induce a hypomethylated state (low DNA methylation) of important target transcripts or loci associated with cellular growth and as a result, lead to enhanced gene expression when exposed to later life anabolic encounters.

To test this hypothesis, we aimed to investigate an epigenetic memory of earlier hypertrophy in adult human skeletal muscle using a within measures design, by investigating genome wide DNA methylation of over 850,000 CpG sites after: (1) Resistance exercise induced muscle growth (loading), followed by; (2) cessation of resistance exercise to return muscle back towards baseline levels (unloading), and; (3) a subsequent later period of resistance exercise induced muscle hypertrophy (reloading). This allowed us to assess the epigenetic regulation of skeletal muscle; (a) hypertrophy, (b) a return of muscle back to baseline and, (c) memory of previous encounters with hypertrophy, respectively. Importantly, these investigations for the first time identified an increased frequency of hypomethylation across the genome during later reloading where lean muscle mass increases were enhanced compared to earlier loading. We also detected genes; AXIN1, GRIK 2, CAMK4 and TRAF1 displayed increasing DNA hypomethylation together with enhanced gene expression across loading, unloading and reloading. Where hypomethylation of these genes was maintained even during unloading where muscle mass returned back to baseline, indicating an epigenetic memory of earlier muscle growth. Furthermore, UBR5, RPL35a, HEG1 and PLA2G16 previously unstudied in skeletal muscle, together with SETD3 displayed hypomethylation and enhanced gene expression following loading versus baseline and displayed even larger increases in both hypomethylation and gene expression after later reloading, also indicating an epigenetically regulated memory leading to enhanced gene expression during reloading. Gene expression of this cluster also strongly and positively correlated with increased muscle mass across all conditions, confirming these transcripts to be novel resistance exercise induced- hypertrophy genes in skeletal muscle. Finally, we identified genes GRIK2, TRAF1 (identified above), BICC1 and STAG1 were hypomethylated after a single bout of acute resistance exercise that were maintained as hypomethylated, and had enhanced gene expression after later reloading. Suggesting that these are epigenetically sensitive genes after a single bout of resistance exercise and associated with enhanced muscle hypertrophy 22 weeks later.

## Methods

### Human Participants and Ethical Approval

Eight healthy males gave written, informed consent to participate in the study, following successful completion of a readiness to exercise questionnaire and a pre-biopsy screening as approved by a physician. One participant withdrew from the study at experimental week 17 of 21, for reasons unrelated to this investigation. However, consent allowed samples to be analysed prior to withdrawal, therefore for this participant, this included all conditions excluding the final reloading condition (for details see below). Ethical approval was granted by the NHS West Midlands Black Country, UK, Research Ethics Committee (NREC approval no. 16/WM/0103), all methods were performed in accordance with the relevant ethical guidelines and regulations.

### Experimental Design

**Figure 1:**
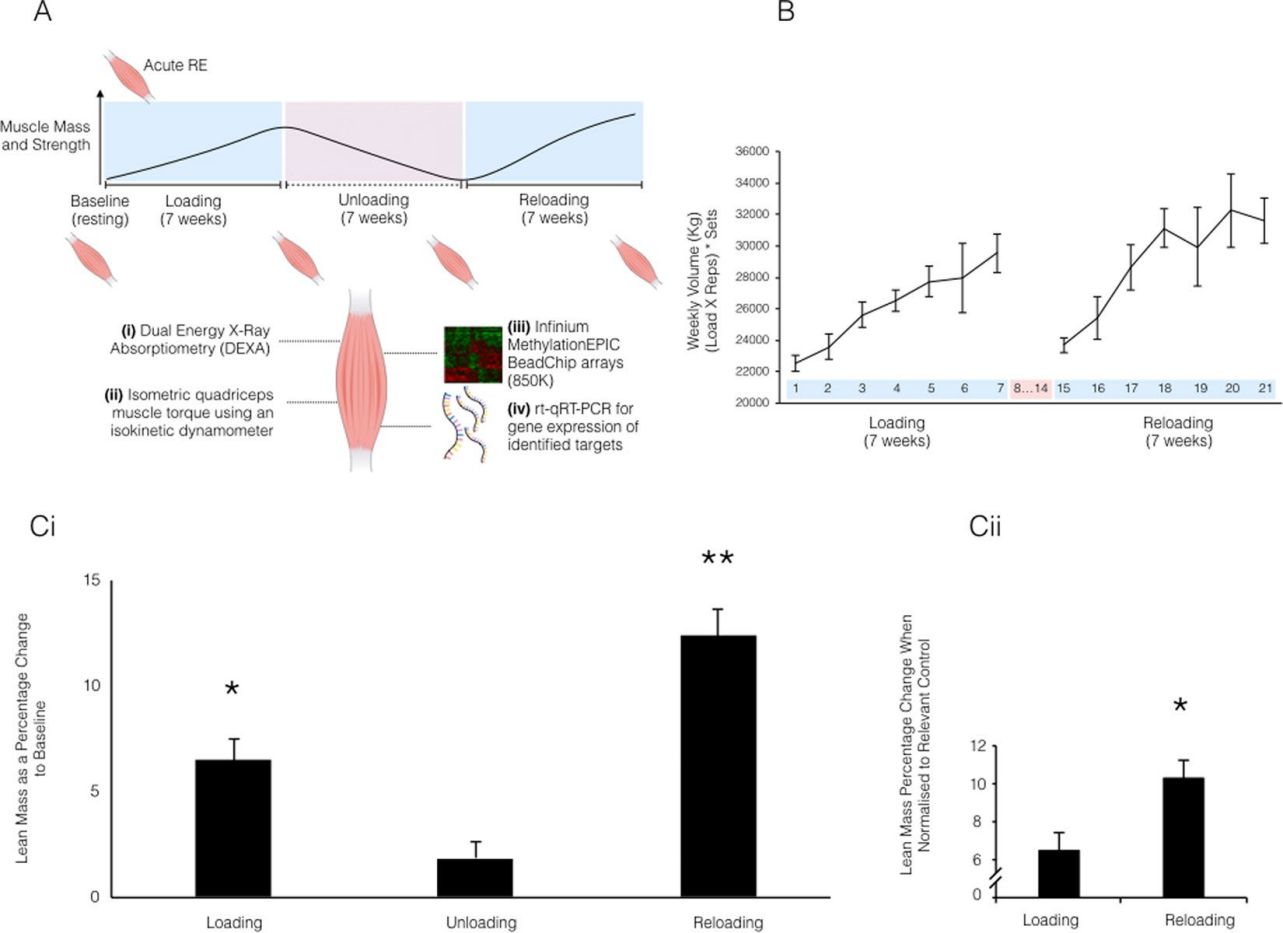
(**A**) Schematic representation of experimental conditions and types of analysis undertaken across the time-course. The image of a muscle represents the time point for analysis of muscle mass via (i) DEXA and strength via (ii) isometric quadriceps muscle torque using an isokinetic dynamometer. The images of muscle tissue also represent the time point of skeletal muscle biopsy of the Vastus Lateralis, muscle sample preparation for downstream analysis of (iii) Infinium MethylationEPIC BeadChip arrays (850 K CpG sites) methylome wide array (iv) and rt-qRT-PCR for gene expression analysis of important genes identified following methylome wide analysis. (**B**) Weekly total volume of resistance exercise undertaken by human participants (n = 7) during the first 7-week resistance exercise period (loading, weeks 1–7), followed by a 7 week cessation of resistance exercise (unloading, weeks 8–14) and the later second period of 7 weeks resistance exercise (reloading, weeks 15–21). Data represents volume load as calculated by ((load (Kg) x reps) x sets)) averaged across 3 resistance exercise sessions per week. Data presented mean ± SEM. (**Ci**) Lean lower limb mass changes in human subjects (n = 7) after a period of 7 weeks resistance exercise (loading), exercise cessation (unloading) and a subsequent second period of 7 weeks resistance exercise (reloading). Total limb lean mass normalised to baseline (percentage change). Significant change compared to baseline represented by * and significant difference to all other conditions represented by ** (**Cii**) Total lean mass percentage change when loading is normalised to baseline, and reloading normalised to unloading to account for starting lean mass in both conditions. Pairwise t-test of significance indicated by *. All data presented as mean ± SEM (n = 7).

Using a within subject design, eight previously untrained male participants (27.6 ± 2.4 yr, 82.5 ± 6.0 kg, 178.1 ± 2.8 cm, means ± SEM) completed an acute bout of resistance exercise (acute RE), followed by 7 weeks (3d/week) of resistance exercise (loading), 7 weeks of exercise cessation (unloading) and a further period of 7 weeks (3d/week) resistance exercise (re-loading). Graphical representation of experimental design is provided in Fig. 1A. Whole-body fan beam dual-energy x-ray absorptiometry (DEXA), strength of the quadriceps via dynamometry and muscle biopsies from the vastus lateralis for RNA and DNA isolation were obtained at baseline, after 7 weeks loading (beginning of week 8), 7 weeks unloading (end of week 14) and 7 weeks reloading (beginning of week 22). A muscle biopsy was also obtained 30 minutes after acute RE prior to 7 weeks loading. Genome-wide analysis of DNA methylation was performed via Illumina EPIC array (850,000 CpG sites- detailed below) for participants across all conditions (n = 8 baseline, acute, loading, unloading, n = 7 reloading). Rt-qRT-PCR was used to investigate corresponding transcript expression of epigenetically altered genes identified via the genome wide DNA methylation analysis.

### Resistance exercise induced muscle hypertrophy: Loading, unloading and reloading

Untrained male subjects initially performed an exercise familiarization week, in which participants performed all exercises with no/low load to become familiar with the exercise type (detailed below). In the final session of the familiarzation week, the load that participants could perform 4 sets of 8–10 repetitions for each exercise was assessed. Due to participants being uncustomized to resistance exercise, assessment was made on competence of lifting technique, range of exercise motion and verbal feedback (participant), and a starting load was set for each participant on an individual basis (mean load for this starting load is included below). Three to four days later, participants then undertook a single bout of lower limb resistance exercise (acute RE, exercises detailed below) followed by biopsies 30 minutes post exercise. Following this single bout of acute RE they then began a chronic resistance exercise program, completing 60-min training sessions (Monday-Wednesday-Friday), for 7 weeks, with 2 sessions/week focusing on lower limb muscle groups (Monday and Friday) and the third session focusing on upper body muscle groups (Wednesday). Lower limb exercises included, behind head squat, leg press, leg extension, leg curl, Nordic curls, weighted lunges and calf raises. Upper limb exercises included, flat barbell bench press, shoulder press, latissimus pull down, dumbbell row and triceps cable extension. To ensure progression in participants with no previous experience in resistance exercise, a progressive volume model was adopted^20^ in which investigators regularly assessed competency of sets, reps and load of all exercises. Briefly, exercises were performed for 4 sets of 10 reps in each set, ~90–120 s in between sets and ~3 mins between exercises. When participants could perform 3 sets of 10 repetitions without assistance and with the correct range of motion, load was increased by ~5–10% in the subsequent set and participants continued on this new load until further modifications were required, as similar to that previously described^20^. Where subjects failed to complete 10 full repetitions (usually for their final sets), they were instructed to reduce the load in order to complete a full repetition range for the subsequent (usually final) set. Total weekly volume load was calculated as the sum of all exercise loads;

$${Total}\,{exercise}\,{volume}\,({kgs})=({Exercise}\,{load}\,({kgs})\ast {No}{.of}\,{Reps})\ast {No}{.Sets}$$

The acute resistance exercise session resulted in a total load of 8,223 kg (± 284 kg). Thereafter, the loading and reloading phases resulted in a progressive increase in training volume (±SEM) of 2,257 ± 639 kg and 2,386 ± 222 kg respectively per week (Fig. 1B), with the reloading phase displaying a significant (P = 0.043) increase in average load. Loading and reloading programs were conducted in an identical manner, with the same exercises, program layout (same exercises on the same day), sets and repetition pattern and rest between sets and exercises. During the 7 week unloading phase, participants were instructed to return to habitual pre-intervention exercise levels and not to perform any resistance training. Regular verbal communication between researcher and participant ensured subjects followed these instructions. A trainer was present at all resistance exercise sessions to enable continued monitoring, provide verbal encouragement and to ensure sufficient progression. No injuries were sustained throughout the exercise intervention.

### Lean mass and strength of the lower limbs by dual-energy x-ray absorptiometry (DEXA) and dynamometry

A whole-body fan beam dual-energy x-ray absorptiometry (DEXA; Hologic QDR Series, Discovery A, Bedford, MA, USA) scan was performed after loading, unloading and reloading (depicted in Fig. 1A) to assess lower limb changes in lean mass. All scans were performed and analysed (QDR for Windows, version 12:4:3) by the same trained operator, according to Hologic guidelines. The DEXA scan was automatically analysed via the QDR software and the operator confirmed areas of interest including lower limb positions. Lean mass was calculated on absolute values for each condition, and presented as percentage change compared to baseline. Furthermore, in addition, a separate analysis was undertaken to assess whether later reloading altered lean mass, where loading was normalised to baseline, and reloading was normalised to unloading to account for any residual starting mass (even if non-significant) following the earlier loading period. A pairwise t-test was then used to analyse the percentage increase in lean mass as a consequence of reloading compared to loading. To assess quadriceps muscle strength, *in-vivo* isometric knee extension maximal voluntary contractions (MVC) were performed using an isokinetic dynamometer (IKD; Biodex, New York, USA) to measure peak joint torque. Data presented as percentage increase to baseline (%) using absolute values (Nm), unless otherwise stated. A full description of strength assessment can be found in Supplementary File 1.

### Muscle Biopsies and Sample Preparation

At baseline, 30 minutes post acute resistance exercise (RE) and after 7 weeks loading (beginning of week 8), 7 weeks unloading (end of week 14) and 7 weeks reloading (beginning of week 22) (Fig. 1A), a conchotome muscle biopsy was obtained from the vastus lateralis muscle of the quadriceps from each participant, avoiding areas of immediate proximity to previous incisions, before being carefully cleaned and dissected using a sterile scalpel on a sterile petri dish. In the unlikely event of any fibrous/fat tissue, this was removed using a scapel, leaving only lean tissue. Separate samples were immediately snap frozen in liquid nitrogen before being stored at −80 °C for RNA and DNA analysis.

### DNA Isolation, Bisulfite Conversion and Methylome Wide BeadChip Arrays

DNA was extracted from frozen tissue samples using a commercially available DNA isolation kit (DNeasy Blood and Tissue Kit, Qiagen, Manchester, UK) in accordance with manufacturer’s instructions, before being analysed (Nanodrop, ThermoFisher Scientific, Paisley, UK) for yield (mean ± SDEV 8.0 µg ± 4.2) and quality (260/280 ratio of mean ± SDEV 1.88 ± 0.09). Five-hundred ng of prepared DNA was bisulfite converted using the EZ-96 DNA Methylation Kit (Zymo Research Corp., CA, USA) following the manufacturer’s instructions for use of the DNA in Illumina assays. Infinium MethylationEPIC BeadChip array examined over 850,000 CpG sites of the human epigenome (Infinium MethylationEPIC BeadChip, Illumina, California, United States) and data was analysed in Partek Genomics Suite V.6.6 (Partek Inc. Missouri, USA). Raw data files (.IDAT) were normalised via the Subset-Quantile Within Array Normalisation (SWAN) method, as previously described^21^. Initial quality control steps were undertaken to detect samples within arrays that were identified as outliers. Principal component analysis (PCA) and normalisation histograms detected two observable outliers across all samples. These samples were removed from any further analysis (Supplementary Figure 1A & B). While skeletal muscle tissue samples may contain a small proportion of other non-muscle cells this analysis suggests sample homogeneity was consistent in the experimental groups and therefore downstream analysis was representative of skeletal muscle tissue and its niche. Data sets represent SWAN-normalised beta (β)-values which correspond to the percentage of methylation at each site and are calculated as a ratio of methylated to unmethylated probes^22^. Differential methylation was subsequently detected across all experimental conditions, and between conditions to identify statistically differentially regulated CpG sites. Fold change in CpG specific DNA methylation and statistical significance was performed using Partek Genomic Suite V.6.6 software, where statistical significance was obtained following ANOVA (with bonferroni correction) analysis.

### Hierarchical Clustering Dendogram

Unadjusted p-value significance (P < 0.05) was used to create a CpG site marker list of standardized beta-values. A standardized expression normalisation was performed to shift CpG sites to mean of zero and scale to a standard deviation of one. Unsupervised hierarchical clustering was performed and dendograms were constructed to represent differentially methylated CpG loci and statistical clustering of experimental samples. Heatmaps represent expression of CpG loci, where reduced methylation at DNA sites (hypomethylated) are represented in green, increased methylation at DNA sites (hypermethylated) in red, and unchanged sites are represented in black.

### Tissue Homogenisation, RNA Isolation and rt-qRT-PCR

Skeletal muscle tissue (~30 mg) was immersed in Tri-Reagent (Sigma-Aldrich, MO, United States) in MagNA Lyser 1.4 mm beaded tubes (MagNA Lyser Green Beads, Roche, Germany) and homogenised for 40 secs at 6 m/s in a MagNA Lyser Homogeniser (Roche, Germany), before being stored on ice for 5 mins. This step was repeated three times to ensure complete disruption of muscle tissue sample. RNA was extracted using standard Tri-Reagent procedure via chloroform/isopropanol extractions and 75% ethanol washing as per manufacturer’s instructions. RNA pellets were resuspended in 30 μl of RNA storage solution (Ambion, Paisley, UK) and analysed (Nanodrop, ThermoFisher Scientific, Paisley, UK) for quantity (mean ± SDEV ; 6671 ± 3986 ng) and an indication of quality (260/280 ratio of mean ± SDEV, 1.95 ± 0.09). For rt-qRT-PCR using QuantiFast^TM^ SYBR^®^ Green RT-PCR one-step kit on a Rotorgene 3000Q, reactions were setup as follows; 9.5 μl experimental sample (5.26 ng/μl totaling 50 ng per reaction), 0.15 μl of both forward and reverse primer of the gene of interest (100 μM), 0.2 μl of QuantiFast RT Mix (Qiagen, Manchester, UK) and 10 μl of QuantiFast SYBR Green RT-PCR Master Mix (Qiagen, Manchester, UK). Reverse transcription was initiated with a hold at 50 °C for 10 minutes (cDNA synthesis), followed by a 5-minute hold at 95 °C (transcriptase inactivation and initial denaturation), before 40–45 PCR cycles of; 95 °C for 10 sec (denaturation) followed by 60 °C for 30 secs (annealing and extension). Primer sequences are provided in Supplementary File 7. Gene expression analysis was performed on at least *n* = 7 for all genes, unless otherwise stated. All relative gene expression was quantified using the comparative Ct (^∆∆^Ct) method. Individual participants own baseline Ct values were used in ^∆∆^Ct equation as the calibrator using RPL13a as the reference gene. The average Ct value for the reference gene was consistent across all participants and experimental conditions (20.48 ± 0.64, SDEV) with low variation of 3.17%.

### Statistical Analysis

Analysis of exercise volume load was performed via a T-test (MiniTab Version 17.2.1) of average participant load during the loading vs. reloading phases. DEXA and isometric peak torque; for *n* = 7, as well as correlation analysis was analysed via a statistical package for the social sciences software for Microsoft (SPSS, version 23.0, SPSS Inc, Chicago, IL) using a one-way repeated measures ANOVA, where applicable. Pearson correlation of coefficient analysis (two tailed) was conducted for gene expression vs. percentage change of leg lean mass. Methylome wide array data sets (*n* = 8 for baseline, acute RE, loading, unloading, *n* = 7 for reloading) were analysed for significant epigenetically modified CpG sites in Partek Genome Suite (version 6.6). All gene ontology and KEGG signalling pathway^23–25^ analysis was performed in Partek Genomic Suite and Partek Pathway, on generated CpG lists of statistical significance (P < 0.05) across conditions (ANOVA) or pairwise comparisons between conditions. In MiniTab Statistical Software (MiniTab Version 17.2.1) follow up rt-qRT-PCR gene expression was analysed using both a MANOVA, to detect for significant interactions across time for identified clusters of genes, and an ANOVA for follow up of individual genes over time. A pairwise t-test was used to analyse gene expression following acute RE vs. baseline. For follow up fold change in CpG DNA methylation analysis was performed via ANOVA in MiniTab Statistical Software (MiniTab Version 17.2.1). Statistical values were considered significant at the level of P ≤ 0.05. All data represented as mean ± SEM unless otherwise stated.

## Results

### Lean leg muscle mass is increased after loading, returns toward baseline during unloading and is further increased after reloading

Analysis of lower limb lean mass via DEXA, identified a significant increase of 6.5% ( ± 1.0%; P = 0.013) in lean mass after 7-wks of chronic loading compared to baseline (20.74 ± 1.11 kg loading vs. 19.47 ± 1.01 kg baseline). Following 7-wks of unloading, lean mass significantly reduced by 4.6% ± 0.6% (P = 0.02) vs. the 7 weeks loading, back towards baseline levels (unloading, 19.83 ± 1.06 kg), confirmed by no significant difference between unloading and baseline. Subsequently, a significant increase in lean mass of the lower limbs was accrued after the reloading phase of 12.4 ± 1.3%, compared to baseline (reloading, 21.85 ± 2.78 kg, P = 0.001, Fig. 1Ci), resulting in an increase of 5.9 ± 1.0% compared to the earlier period of loading (P = 0.005). Pairwise t-test analysis that corrected for any lean mass that was maintained during unloading demonstrated a significant increase in lean muscle mass in the reloading phase (unloading to reloading), compared to the loading phase (baseline to loading) (P = 0.022; Fig. 1Cii). Analysis of muscle strength suggested a similar trend. Isometric peak torque increased by 9.3 ± 3.5% from 296.2 ± 22.1 Nm at baseline to 324.5 ± 27.3 Nm after 7-wks of loading, this difference was not statistically significant (Supplementary Figure 2). Upon 7-wks of unloading, peak torque reduced by 8.3 ± 2.8% vs. loading, back towards baseline levels. Upon subsequent reloading, a significant 18 ± 3.6% increase in isometric peak torque production (349.6 ± 27.7 Nm) was observed compared to baseline (P = 0.015; Supplementary Figure 2A).

### The largest DNA hypomethylation across the genome occurred following reloading

**Figure 2:**
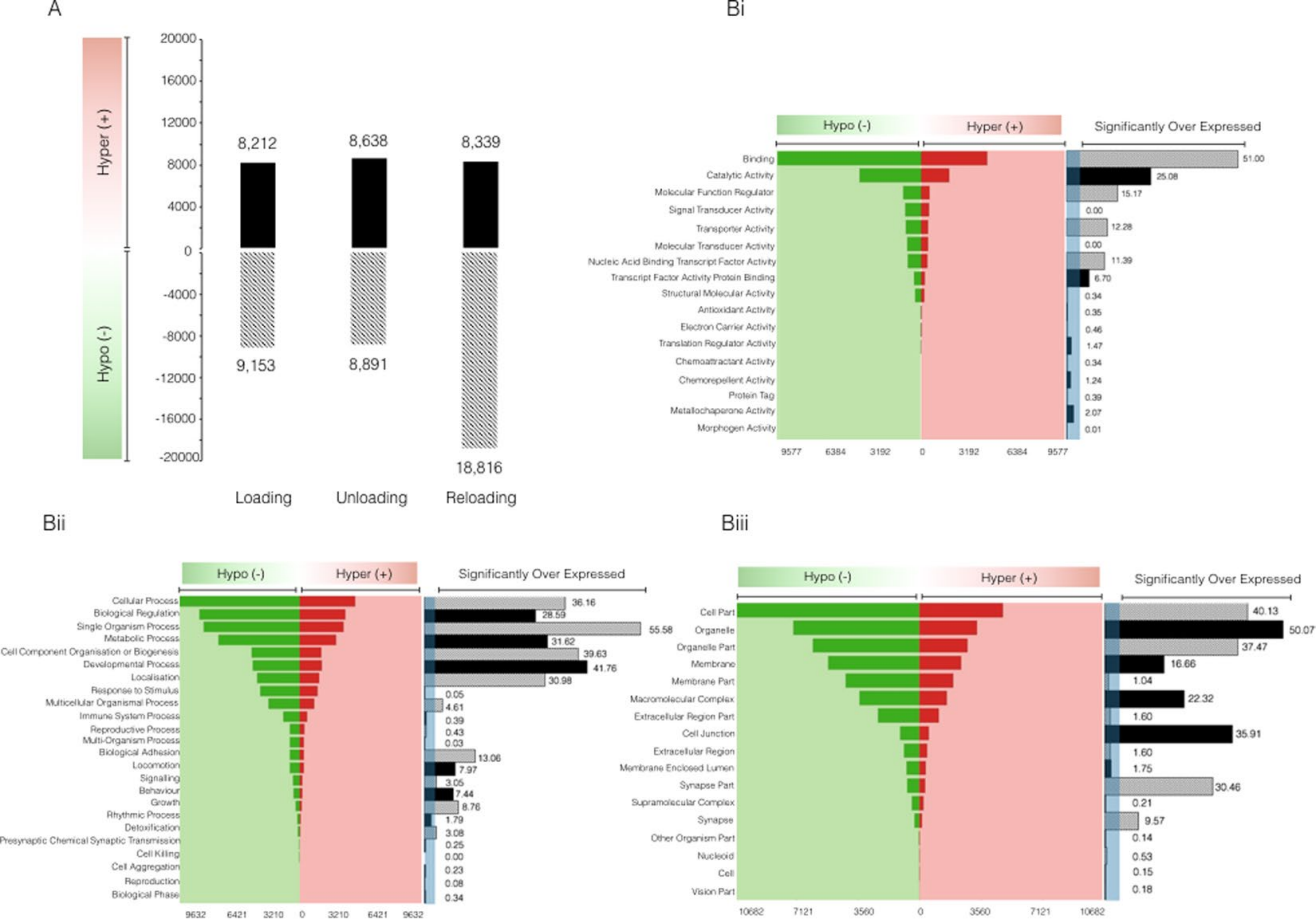
(**A**) Infinium MethylationEPIC BeadChip arrays (850 K CpG sites) identified an enhanced frequency of hypomethylated CpG sites upon reloading (n = 7). (**B**) Gene ontology analysis using forest plot schematics confirmed an enhanced hypomethylated profile after reloading across various (**i**) molecular function, (**ii**) biological processes and (**iii**) cellular components. Functional groups with a fold enrichment >3 (as indicated via shaded blue region) represents statistically ‘over expressed’ (in this case epigenetically modified) KEGG pathways FDR < 0.05 (n = 8).

The frequency of statistically (P* < *0.05) differentially regulated CpGs in each condition was analysed (Fig. 2A; Supplementary File 2B). 17,365 CpG sites were significantly (P* < *0.05) differentially epigenetically modified following loading induced hypertrophy compared to baseline, with a larger number being hypomethylated (9,153) compared to hypermethylated (8,212) (Fig. 2A; Supplementary File 2A & B). The frequency of hypomethylated epigenetic modifications was similar to loading after unloading (8,891) (Fig. 2A; Supplementary File 2A & C), where we reported lean muscle mass returned back towards baseline. Importantly, following reloading induced muscle growth we observed an increase in the number of epigenetically modified sites (27,155) and an enhanced number of hypomethylated DNA sites (18,816, Fig. 2A; Supplementary File 2A & D). This increase in hypomethylation coincided with the largest increase in skeletal muscle mass in reloading. By contrast, hypermethylation remained stable (8,339) versus unloading (8,638) and initial loading (8,212). To further analyse the reported increased frequency of hypomethylated genes across the genome following reloading, gene ontologies were analysed for the frequency of hypo and hypermethylated CpG sites. In agreement with our above frequency analysis, the most statistically significant enriched GO terms identified an increased number of hypomethylated CpG sites compared to baseline (Fig. 2Bi–iii). Indeed, the most statistically significantly (FDR < 0.05) enriched GO terms were: 1) molecular function GO:0005488 encoding for genes related to ‘binding’, that displayed 9,577 (68.71%) CpG sites that were hypomethylated following reloading and 4,361 (31.29%) sites as hypermethylated compared to baseline (Fig. 2Bi), and: 2) Biological process GO:0044699 encoding for genes related to ‘single-organism processes’ that displayed 7,586 (68.57%) hypomethylated CpG sites compared to 3,493 (31.43%) sites profiled as hypermethylated after reloading compared to baseline (Fig. 2Bii). Finally, 3) cellular component, GO:004326 encoding for genes related to ‘organelle’ reported 7,301 hypomethylated CpG sites following reloading and 3,311 hypermethylated sites, compared to baseline, therefore favouring a majority 68.88% hypomethylated profile (Fig. 2Biii).

**Figure 3:**
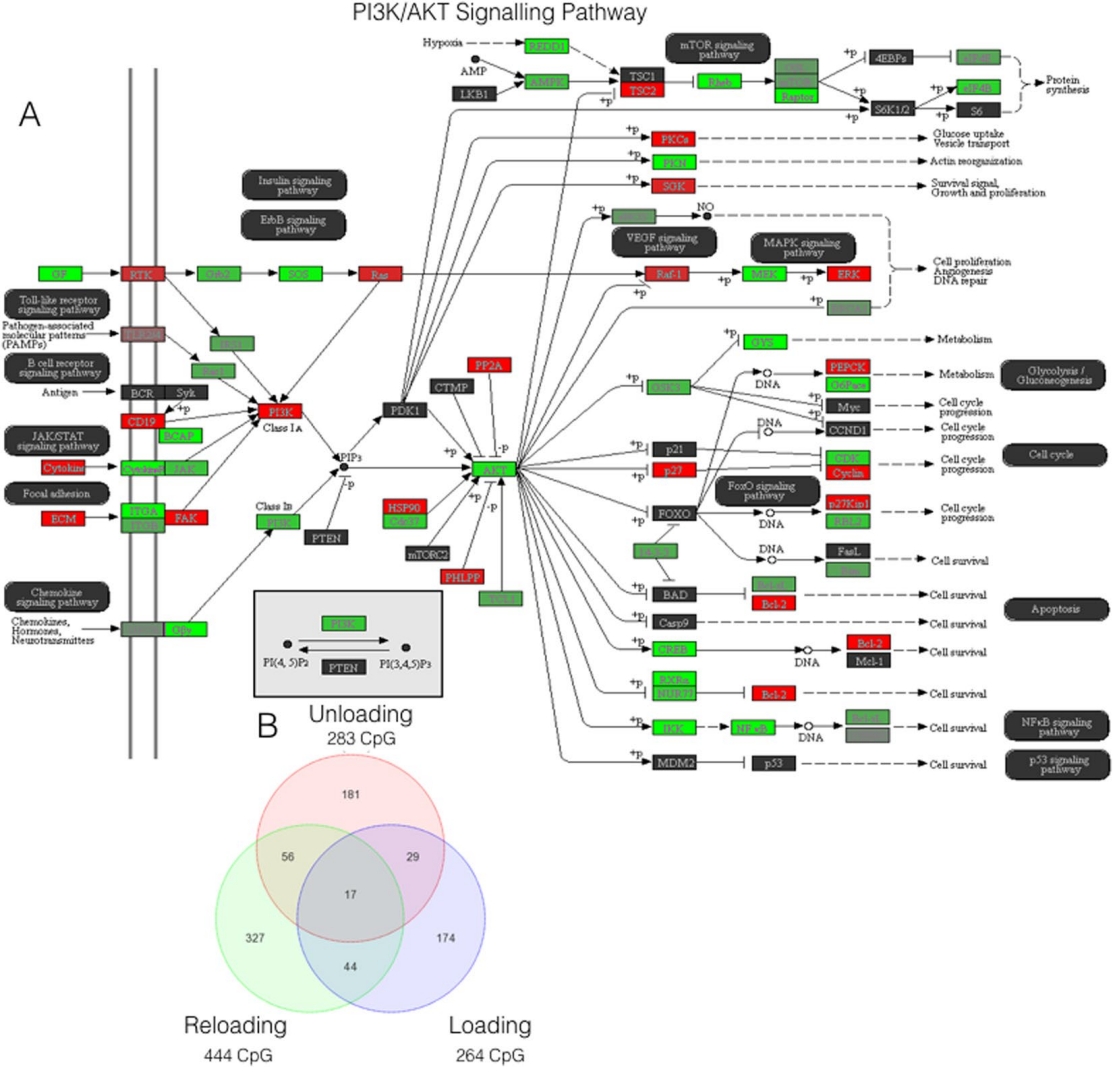
(**A**) Representation of the DNA methylation modifications that occurred within the PI3K/AKT KEGG pathway following 7 weeks of reloading in human subjects. Signalling analysis performed on statistically differentially regulated CpG sites compared to baseline, with green indicating a hypomethylated fold change and red indicating a hypermethylated change, with strength of colour representing the intensity of fold change^23–25^. Figure 3b. Venn diagram analysis of the statistically differentially regulated CpG sites attributed to the PI3K/AKT pathway following loading, unloading and reloading, compared to be baseline. Ellipsis reports number of commonly statistically differentially regulated CpG sites across each condition. Analysis confirms an enhanced number of differentially regulated CpG sites upon reloading condition.

Following confirmation that the largest alteration in CpG DNA methylation occurred upon later reloading evoked hypertrophy , we sought to elucidate how the serine/threonine AKT signaling pathway, a critical pathway involved in mammalian growth, proliferation and protein synthesis^26,27^, was differentially regulated across experimental conditions (Fig. 3, Supplementary Figure 3A and B). Intuitively, we report that the PI3K/AKT pathway was significantly enriched upon all pairwise comparisons of baseline vs. loading, unloading and reloading, respectively (P < 0.022; Supplementary Figure 3A, B and Fig. 3A), suggesting that the pathway was significantly epigenetically modified following periods of skeletal muscle perturbation. Importantly, frequency analysis of statistically differentially regulated transcripts (Fig. 3B) attributed to this pathway, reported an enhanced number of differentially regulated CpG sites (444 CpG sites) following reloading (Fig. 3A), compared to loading ( 264 CpG sites; Supplementary Figure 3A) and unloading (283 CpG sites; Supplementary Figure 3B) alone. In accordance with our previous findings, the enhanced number of statistically differentially regulated CpG sites in this pathway upon reloading is attributed to an enhanced number of hypomethylated (299 CpG sites, 67.3%) compared to hypermethylated (145 sites, 32.7%) CpG sites (Raw data: Supplementary File 3).

### Genome-wide DNA methylation analysis identified two clusters of temporal DNA methylation patterns that provide initial evidence of an epigenetic memory

**Figure 4:**
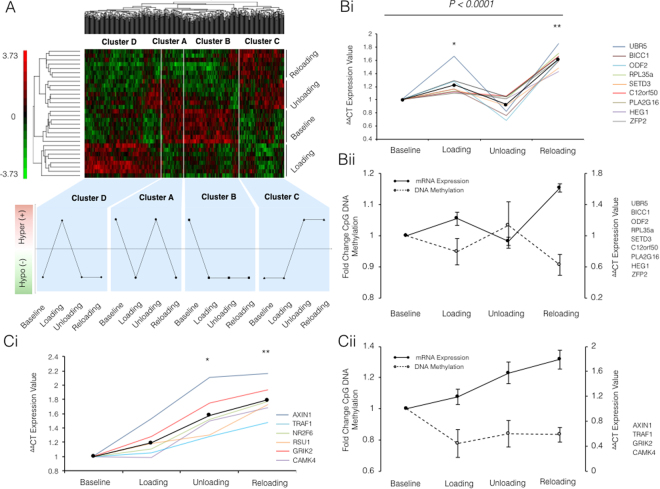
(**A**) Heat map depicting unsupervised hierarchical clustering of the top 500 statistically differentially regulated CpG loci (columns) and conditions (baseline, loading, unloading and reloading) in previously untrained male participants (n = 8). The heat-map colours correspond to standardised expression normalised β-values, with green representing hypomethylation, red hypermethylation and unchanged sites are represented in black. (**4B** and **C**) Relative gene expression (i) and schematic representation of CpG DNA methylation and gene expression relationship (ii) in two identified gene clusters from genome wide methylation analysis after a period of 7 weeks resistance exercise (loading), exercise cessation (unloading) and a subsequent secondary period of 7 weeks resistance exercise (reloading). (**Bi**) Expression of genes that displayed a significant increase compared to baseline (represented by *) upon earlier loading, that returned to baseline during unloading, and displayed enhanced expression after reloading (significantly different to all other conditions **). MANOVA analysis reported a significant effect over the entire time course of the experiment (P < 0.0001). (**Bii**) Representative schematic displaying the inverse relationship between mean gene expression (solid black lines) and CpG DNA methylation (dashed black lines) of grouped transcripts (RPL35a, C12orf50, BICC1, ZFP2, UBR5, HEG1, PLA2G16, SETD3 and ODF2). Data represented as fold change for DNA methylation (left y axis) and gene/mRNA expression (right y axis). (**Ci**) Clustering of genes that portrayed an accumulative increase in gene expression after loading, unloading and reloading. With the largest increase in gene expression after reloading. Culminating in significance in the unloading (baseline vs. unloading*), and reloading (reloading vs. baseline**). (**Cii**) Representative schematic displaying the inverse relationship between mean gene expression (solid black lines) and CpG DNA methylation (dashed black lines) of grouped transcripts (AXIN1, TRAF1, GRIK2, CAMK4). Data represented as fold change for methylation (left y axis) and mRNA expression (right y axis). All data represented as mean ± SEM for gene expression (n = 7 for UBR5, PLA2G16, AXIN1, GRIK2; n = 8 for all others) and CpG DNA methylation (n = 8 for baseline, loading and unloading; n = 7 for reloading).

Changes in genome-wide DNA methylation were analysed following loading, unloading and reloading induced muscle adaptation. A dendogram of the top 500 most statistically epigenetically modified CpG sites across each experimental condition compared to baseline, identified large alterations in DNA methylation profiles (Fig. 4A; Supplementary File 4A). A ranked unsupervised hierarchical clustering analysis demonstrated significant differences between the initial loading (weeks 1–7) vs. all other conditions (Fig. 4A). Closer analysis of the top 500 CpG sites across experimental conditions highlighted a clear temporal trend occurring within different gene clusters. The first cluster (named Cluster, A) displayed enhanced hypomethylation with earlier loading-induced hypertrophy. This cluster was methylated at baseline and became hypomethylated after loading, re-methylated with unloading (Fig. 4A) and hypomethylated after reloading. The second temporal trend (named Cluster B) also displayed an enhanced hypomethylated state across the top 500 CpG sites as a result of load induced hypertrophy. As with Cluster A, Cluster B genes were methylated at baseline and became hypomethylated after initial loading. In contrast to Cluster A, Cluster B remained hypomethylated with unloading, even when muscle returned to baseline levels, and this hypomethylation was also maintained/‘remembered’ after reload induced hypertrophy (Cluster B, depicted Fig. 4A). The third temporal trend, named Cluster C, revealed genes as hypomethylated at both baseline and after initial loading, suggesting no epigenetic modification after the first period of hypertrophy in these genes (Cluster C, Fig. 4A). During unloading, genes were hypermethylated and remained in this state during reloading. The final cluster (Cluster D) of genes, were hypomethylated at baseline, became hypermethylated after loading (Cluster D, Fig. 4A), reverted back to a hypomethylated state with unloading and then maintained the hypomethylated state after reloading, reflecting the profile of the baseline targets in the same cluster (Cluster D, Fig. 4A). These two clusters (C&D) did report a maintenance of the DNA methylation profile from unloading to reloading conditions. Cluster C also reported a hypermethylated profile after unloading following a period of loading, that may therefore identify important CpG sites that are hypermethylated when muscle mass is reduced (we therefore include a full list from cluster C that includes the CpG sites significantly modified in loading vs. unloading, Supplementary File 4G). However, both Cluster C&D suggest no retention of epigenetic modifications from the first loading period to the later reloading phase.

### Identification of gene expression clusters inversely associated with DNA methylation

To assess whether the changes in DNA methylation affected gene expression, the 100 most significantly differentially modified CpG sites across all conditions were identified and cross referenced with the most frequently occurring (Supplementary File 4B) CpG modifications in pairwise comparisons of all conditions (Supplementary File 4C to H). This identified 48 genes that were then analysed by rt-qRT-PCR to assess gene expression. Forty-six percent of the top 100 CpG sites were within gene promotor regions with 18% residing in intergenic regions (Supplementary File 5). Interestingly, gene expression analysis identified two distinct clusters of genes that had different transcript profiles. This first cluster included RPL35a, C12orf50, BICC1, ZFP2, UBR5, HEG1, PLA2G16, SETD3 and ODF2 genes that displayed a significant main effect for time (P < 0.0001) after MANOVA analysis (Fig. 4Bi). Chromosome locations, reference sequence numbers and gene region section details for these genes can be found in Supplementary File 5. Importantly, this first cluster displayed a mirrored (inverse) temporal pattern to those identified previously in Cluster A above for CpG methylation (in the top 500 differentially regulated CpG sites, Fig. 4A). Where, upon 7-wks of loading, gene expression of this cluster significantly increased (1.22 ± 0.09, P = 0.004) and CpG methylation of the same genes was non-significantly reduced (hypomethylated) (0.95 ± 0.04 Fig. 4Bii). During unloading, methylation returned to baseline (1.03 ± 0.07), which was met by a return to baseline in gene expression (0.93 ± 0.05), as indicated by both CpG methylation and gene expression displaying no significant difference compared to baseline (Fig. 4Bii). Importantly, upon reloading, both CpG methylation and gene expression displayed an enhanced response compared to the baseline and loading time point, respectively. Indeed, upon reloading, this cluster became hypomethylated (0.91 ± 0.03, P = 0.05, Fig. 4Bii). This was met with a significant enhancement (1.61 ± 0.06) in gene expression of the same cluster compared to baseline and loading (P < 0.001, Fig. 4Bii).

A second separate gene cluster was identified and included: AXIN1, GRIK2, CAMK4, TRAF1, NR2F6 and RSU1. Although together there was no significant effect of time via MANOVA analysis. ANOVA analysis reported that this cluster displayed increased gene expression after loading (1.19 ± 0.08) that then further increased during unloading (1.58 ± 0.13) resulting in statistical significance (P = 0.001) compared to baseline alone. Gene expression was then even further enhanced (1.79 ± 0.09) upon reload induced hypertrophy (P < 0.0001; Fig. 4Ci; Chromosome locations, reference sequence numbers, region section details for this cluster of genes can be found in Supplementary File 5). In this cluster we identified an accumulative increase in gene expression, attaining significance at unloading condition (ANOVA; P = 0.001) compared to baseline, gene expression was subsequently further increased following reloading conditions (ANOVA; P < 0.0001). This temporal gene expression pattern was inversely associated to CpG methylation observed in Cluster B (identified previously in the top 500 differentially regulated CpG sites, Fig. 4A). Closer fold-change analysis of CpG DNA methylation of this gene cluster, identified a distinct inverse relationship with methylation and gene expression of 4 out of 6 of the targets (AXIN1, GRIK2, CAMK4, TRAF1). Where, upon loading, these genes became significantly hypomethylated (0.78 ± 0.09; P = 0.036) compared to baseline, with this profile being maintained during unloading (0.84 ± 0.09) and reloading (0.83 ± 0.05) conditions, albeit non-significantly. Collectively, we report that a sustained hypomethylated state in 4 out of 6 of the genes in this cluster that correspond to an increased transcript expression of the same genes (Fig. 4Cii).

### Identification of a number of novel genes at the expression level associated with skeletal muscle hypertrophy

**Figure 5:**
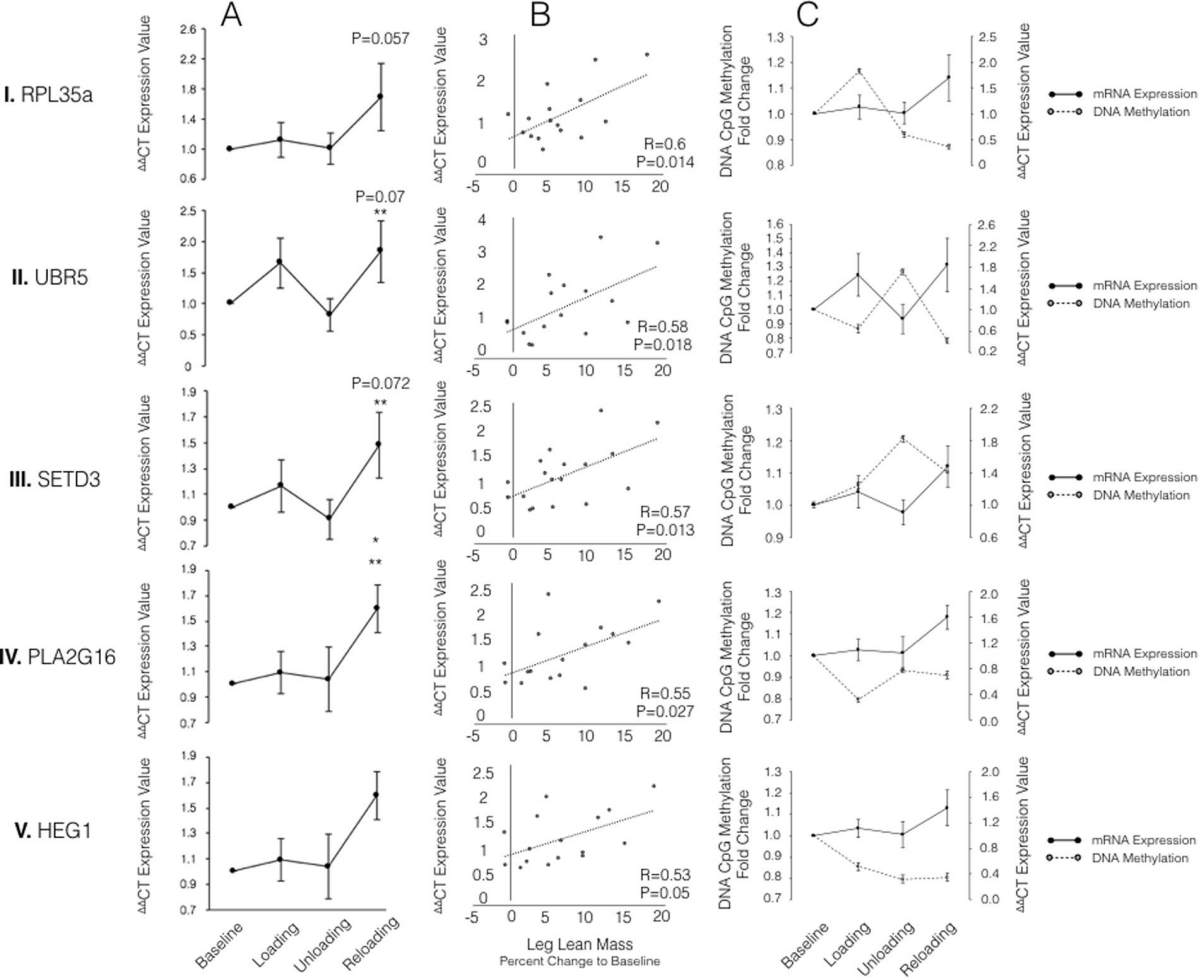
Relative fold changes in: (**A**) gene expression; (**B**) correlation between gene expression and lower limb lean mass across experimental conditions, and; (**C**) schematic representation of relationship between fold changes in CpG DNA methylation (dashed black line; left y axis) and fold change in gene/mRNA expression (solid black line; right y axis) for identified genes: RPL35a (I), UBR5 (II), SETD3 (III), PLA2G16 (IV) and HEG1 (V). Statistical significance compared to baseline and unloading represented by* and** respectively. All significance taken as p less than or equal to 0.05 unless otherwise state on graph. All data presented as mean ± SEM (n = 7/8).

To ascertain the relationship between skeletal muscle hypertrophy and gene expression, fold change in gene expression was plotted against percentage changes (to baseline) in leg lean mass. Interestingly, in our first cluster of genes identified above (RPL35a, C12orf50, BICC1, ZFP2, UBR5, HEG1, PLA2G16, SETD3 and ODF2), a significant correlation between gene expression and lean mass was observed for genes RPL35a, UBR5, SETD3, PLA2G16 and HEG1 (Fig. 5A & BI–V). Following exposure to 7-wks of load induced hypertrophy, RPL35a gene expression displayed a non-significant increase compared to baseline (1.13 ± 0.23; Fig. 5AI), that upon unloading returned back to the baseline levels (1.01 ± 0.21). Upon reloading, the expression of RPL35a increased to 1.70 ( ± 0.44; Fig. 5AI) compared to baseline (P = 0.05). This expression pattern across loading, unloading and reloading conditions corresponded to a significant correlation with percentage changes in skeletal muscle mass (R = 0.6, P = 0.014; Fig. 5BI), with RPL35a accounting for 36% of the variation in muscle across experimental conditions. Both UBR5 and SETD3 displayed similar percentage accountability for the change in skeletal muscle mass across conditions. Indeed, UBR5 and SETD3 accounted for 33.64% and 32.49% of the variability in skeletal muscle mass, respectively, both portraying strong correlations between their gene expression and the percentage change in lean leg mass (UBR5, R = 0.58, P = 0.018, Fig. 5BII; SETD3, R = 0.57, P = 0.013, Fig. 5BII, respectively). Additionally, UBR5 (1.65 ± 0.4; Fig. 5BII) and SETD3 (1.16 ± 0.2; Fig. 5AIII) both demonstrated non-significant increases in gene expression after 7-wks of loading (P > 0.05), with the expression of both genes, UBR5 (0.82 ± 0.27) and SETD3 (0.90 ± 0.15), returning to baseline levels upon 7-wks of unloading (Fig. 5AII and AIII, respectively). Furthermore, upon reloading UBR5 displayed its greatest increase in expression (1.84 ± 0.5; Fig. 5AII), demonstrating a trend for significance compared to baseline condition (P = 0.07), and a significant increase compared to unloading (P = 0.035). Whereas, SETD3 demonstrated a fold increase of 1.48 ( ± 0.25; Fig. 5AIII) approaching significance compared to baseline (P = 0.072) and achieving significance compared to unloading (P = 0.036). PLA2G16 also demonstrated a significant correlation between its fold change in gene expression and the percentage change in skeletal muscle mass (R = 0.55; P = 0.027; Fig. 5BIV), with PLA2G16 accounting for 30.25% of the change in skeletal muscle. Interestingly, across conditions, PLA2G16 demonstrated the greatest significant changes in gene expression. Indeed, loading induced hypertrophy, PLA2G16 displayed a non-significant increase compared to baseline in expression (1.09 ± 0.17; Fig. 5AIV), that upon unloading returned back to the baseline levels (1.04 ± 0.25). Importantly, upon reloading, the expression of PLA2G16 significantly increased (1.60 ± 0.18; Fig. 5AIV) compared to baseline (P = 0.026) and unloading conditions (P = 0.046), as well as approaching a significant increase compared to the initial loading stimulus (P = 0.067 compared to load; Fig. 5AIV). HEG 1 gene expression exhibited a significant correlation with skeletal muscle mass (R = 0.53, P = 0.05) with HEG 1 accounting for 28.09% of the changes in muscle mass. However, HEG1 did not demonstrate any significant fold changes in gene expression across the experimental conditions. Furthermore, no significant correlation was observed for the other identified cluster of genes (AXIN1, GRIK2, CAMK4, TRAF1, NR2F6 and RSU1; P > 0.05; Data not shown). Collectively, these data suggest that RPL35a, UBR5, SETD3 and PLA2G16 all display a significantly enhanced gene expression upon reloading induced hypertrophy. This suggests, that these genes portray a memory of earlier load induced hypertrophy, by displaying the largest fold increases in gene expression after reload induced growth.

### The E3 Ubiquitin Ligase, UBR5, has enhanced hypomethylation and the largest increase in gene expression during  reloading

**Figure 6:**
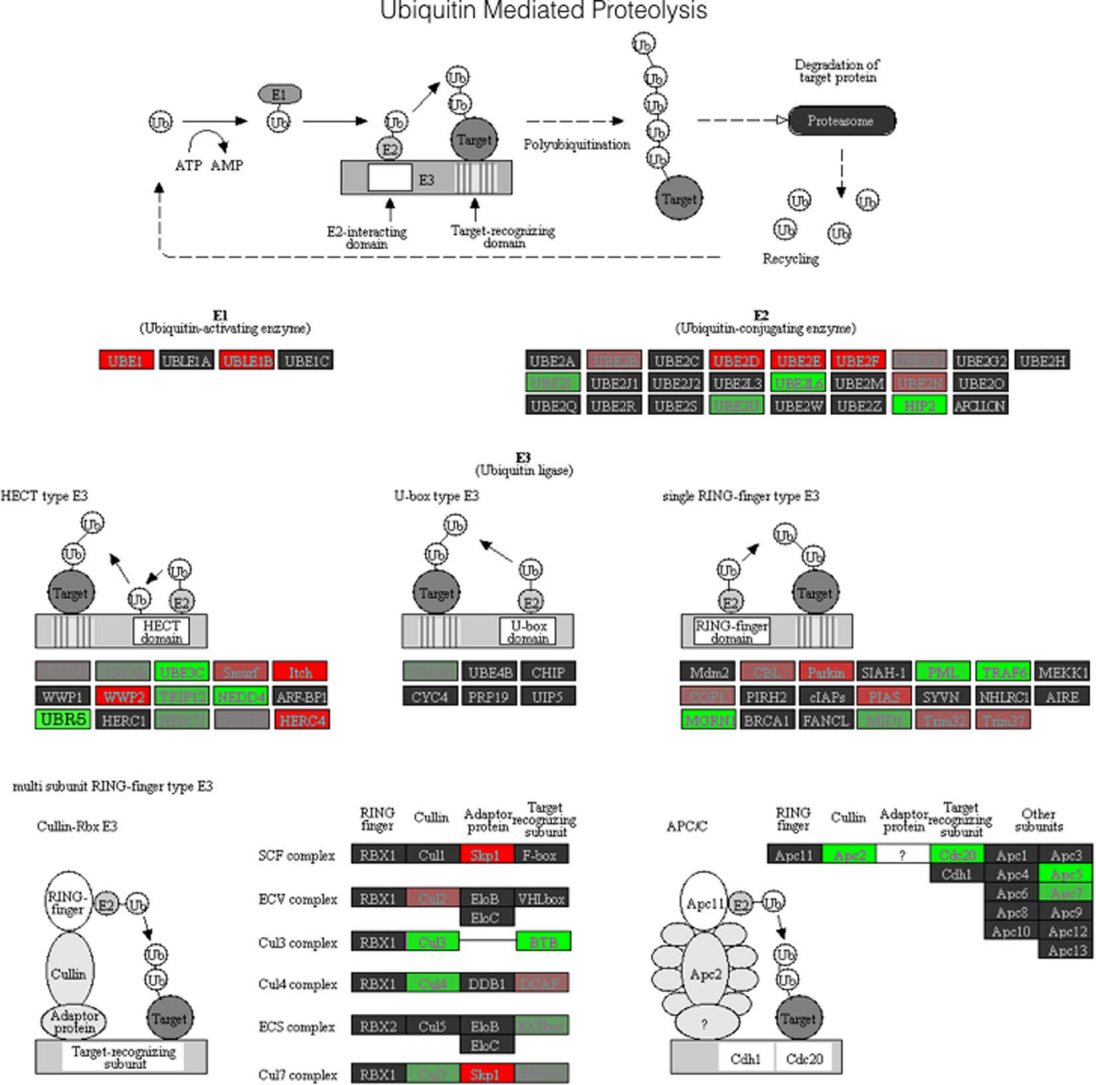
Representation and characterisation of the DNA methylation modifications that occurred within the ubiquitin mediated proteolysis pathway across all conditions of loading, unloading and reloading compared to baseline (ANOVA). Signalling analysis performed on statistically differentially regulated CpG sites compared to baseline, with green indicating a hypomethylated fold change and red indicating a hypermethylated change, with strength of colour representing the intensity of fold change^23–25^. Importantly, the novel HECT-type E3 ubiquitin ligase, UBR5, displays a significantly hypomethylated state within this pathway.

The HECT E3 ubiquitin ligase gene UBR5 (Fig. 6), for which the CpG identified is located on chromosome 8 (start 103424372) in the promoter region 546 bp from the transcription start site, was identified as being within the top 100 most statistically differentially regulated CpG sites across all pair-wise conditions (loading, unloading and reloading; Fig. 6); but also the transcript that displayed the most distinctive mirrored-inverse relationship with gene expression (Fig. 5CII), after every condition. Following the initial period of 7-weeks of load induced hypertrophy, there was a non-significant increase in UBR5 gene expression (1.65 ± 0.4) versus baseline, which was met with a concomitant (albeit non-significant) reduction in CpG DNA methylation (0.87 ± 0.03). Gene expression returned to baseline control levels after unloading (0.82 ± 0.27) demonstrated by a significant reduction vs. loading (P = 0.05) and non-significance versus baseline (P = N.S; Fig. 5CII). After the same unloading condition, we observed a significant increase in CpG DNA methylation compared to baseline (1.27 ± 0.02; P = 0.013; Fig. 5CII). Importantly, upon reloading, UBR5 displayed its largest increase in transcript expression, significantly greater compared to unloading (1.84 ± 0.5 vs. 0.82 ± 0.27, P = 0.035) and versus baseline levels to the level of P = 0.07. Concomitantly, after the reloading condition, we observed the largest statistically significant reduction in CpG DNA methylation (0.78 ± 0.02) compared to baseline (P = 0.039), and unloading (P ≤ 0.05; Fig. 5CII).

### Dynamic changes in DNA methylation after a single acute bout of resistance exercise precede changes in gene expression after loading and reloading

**Figure 7:**
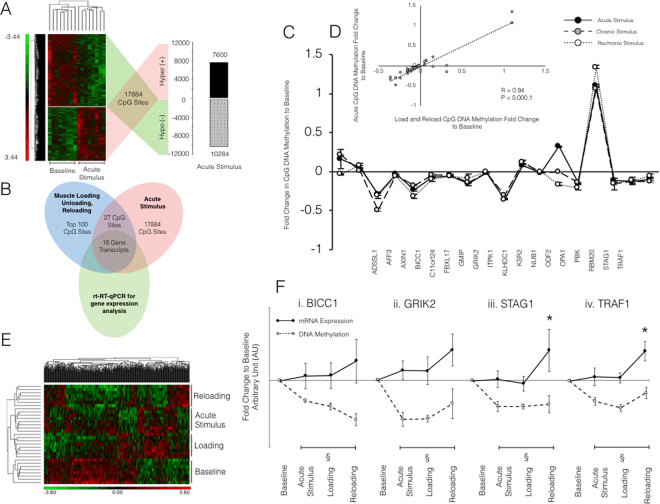
Response of the methylome after acute resistance loading stimulus compared to baseline, 7 weeks loading and 7 weeks reloading: (**A**) Heat map depicting unsupervised hierarchical clustering of statistically differentially regulated (P = 0.05) CpG loci following exposure to acute RE compared to baseline; (**B**) a Venn diagram depicting the number of CpG sites that were significantly differentially regulated in both methylome analysis experiments (base, loading, unloading and reloading, blue circle; baseline and acute resistance stimulus, red circle), and the amount of genes analysed for gene expression across acute, 7 weeks loading and 7 weeks reloading, respectively; (**C**) temporal pattern of fold change in DNA CpG methylation of the identified overlapping CpG sites that mapped to relevant gene transcripts; (**D**) correlation of CpG DNA methylation of acute RE vs. 7 weeks loading and reloading conditions, and (**E**) Heat map depicting unsupervised hierarchical clustering of statistically differentially regulated (P = 0.05) CpG loci following exposure to acute RE compared to baseline, loading and reloading (**F**) representative schematic displaying the inverse relationship between mean gene expression (solid black lines) and CpG DNA methylation (dashed black lines) of identified transcripts. Significance indicated in gene expression (*) and in CpG DNA methylation (§) when compared to baseline.

We next wished to ascertain how dynamic and transient DNA methylation of the identified genes were, after a single acute bout of resistance exercise (acute RE). We wanted to identify methylation sensitive genes (to single acute resistance loading stimuli) that were still affected at the DNA methylation and gene expression levels after later chronic load and reload induced hypertrophy conditions. We identified that acute loading evoked a greater hypomethylation compared to hypermethylation response of the human methylome (10,284 hypomethylated sites vs. 7,600 hypermethylated DNA sites; Fig. 7A) with hierarchical clustering analyses displaying distinct differences between statistically significant CpG sites at baseline and acute RE conditions (P < 0.05; total of 17884 CpG sites, Fig. 7A). This occurred with a similar frequency versus loading where we previously reported 9,153 hypomethylated vs. 8,212 hypermethylated (8,212) CpG sites (Fig. 2A). Overlapping the top 100 significantly differentially identified targets from the loading, unloading and reloading analysis (Supplementary File 4A) together with the 17,884 sites from acute stimulus analysis (Supplementary File 6), identified 27 CpG targets that were significantly differentially regulated across comparisons (Fig. 7B). We subsequently removed 9 CpG sites that did not map to gene transcripts and were therefore unable to analyse for corresponding gene expression. We identified that the fold change in DNA methylation pattern of the remaining 18 CpG sites was virtually identical across these conditions (Fig. 7C), displaying a significant correlation across acute RE to loading and reloading conditions (R = 0.94, P < 0.0001; Fig. 7D), with follow up broader hierarchical clustering analysis of the top 500 genes significantly modified within these conditions (Fig. 7E) also confirming that the majority of sites in were hypomethylated. Suggesting that even after a single bout of acute resistance exercise that the DNA methylation remained the same after later  load and reload induced hypertrophy. Interestingly, we identified 4 of the 18 CpG sites identified above (BICC1, GRIK2, ODF2, TRAF1) that were also identified in our earlier analyses of loading, unloading and reloading conditions (Figs. 7A and B). This suggested that these genes were immediately altered following acute RE, and hypomethylation was retained during chronic loading, unloading and subsequent reloading conditions. Finally, we analysed fold changes in gene expression of a sub set of the 18 CpG sites identified as overlapping in both sets of methylome analysis experiments (Supplementary Figure 4a) and compared changes in gene expression to changes in CpG DNA methylation (Supplementary Figure 4B). We identified that significant hypomethylation upon acute resistance exercise (Figure 7Fi–vii) was not associated with significant changes in gene expression (Figure 7Fi–vii) in a sub-set of analysed transcripts. However, upon continued loading (chronic loading and reloading conditions), changes in CpG DNA methylation were associated with significant changes in a number of these genes upon the reloading stimulus (Figure 7Fi–vii). Suggesting that these newly identified epigenetically regulated genes (BICC1, GRIK2, TRAF1 and STAG1) were acutely sensitive to hypomethylation after a single bout of resistance exercise, that enhanced gene expression 22 weeks after a period of load induced hypertrophy, a return of muscle to baseline and later reloading induced hypertrophy. Therefore, the epigenetic regulation of these genes seems to be an early, acute exercise biomarker of later muscle hypertrophy.

## Discussion

### Frequency of genome-wide hypomethylation is the largest after reloading induced hypertrophy where lean muscle mass is enhanced

We aimed to investigate an epigenetic memory of earlier hypertrophy in adult human skeletal muscle using a within measures design, by undertaking: (1) resistance exercise induced muscle growth (loading), followed by; (2) cessation of resistance exercise, to return muscle back towards baseline levels (unloading), and; (3) a subsequent later period of resistance exercise induced muscle hypertrophy (reloading). We first confirmed that we were able to elicit an increase in lean mass of the lower limbs after 7 weeks loading, that returned back to baseline levels after 7 weeks unloading, with 7 weeks reloading evoking the largest increase in lean mass. Interestingly, after DNA methylation analysis of over 850,000 CpG sites, we identified the largest frequency of hypomethylation (18,816 CpG sites) occurred after reloading where the largest lean mass occurred. Previous studies have suggested that hypermethylation of over 6,500 genes are retained, after an more acute stress of high fat intake (for 5 days) 8 weeks later despite removal of the high fat diet^14^, and hypermethylation occurs following early life inflammatory stress in muscle cells and is maintained for over 30 cellular divisions^11^. The present study also suggested hypomethylation was maintained during unloading (8,891 CpG sites) where muscle mass returned to baseline having being subjected to an earlier period of load induced muscle growth (9,153 CpG sites), then upon reloading the frequency of hypomethylation was enhanced in association with the largest increases in lean mass. Furthermore, bioinformatic analysis of the PI3K/AKT pathway across loading, unloading and reloading conditions, supports the findings of an enhanced hypomethylated state upon secondary exposure to resistance stimulus. Importantly, this pathway is identified as critical for cell proliferation/differentiation, muscle protein synthesis and therefore muscle hypertrophy^27^, and therefore, it is plausible that the enhanced hypomethylated state of the genes in this pathways would lead to enhanced gene expression and protein levels. However, further analysis is required to investigate the total protein or activity of these pathways in this model. Nonetheless, collectively, these results provide initial evidence for a maintenance/memory of universal hypomethylation. The only other study to demonstrate a memory of prior hypertrophy in skeletal muscle was in rodents following earlier encounters with testosterone administration, where a retention of myonuclei occurred even during testosterone withdrawal and a return of muscle to baseline levels^13^, suggesting a memory at the cellular level. However, these are the first studies to demonstrate that a memory occurs at the epigenetic level within skeletal muscle tissue.

### Hypomethylation is maintained from earlier load induced hypertrophy even during unloading where muscle mass returns back towards baseline and is inversely associated with gene expression

Following the frequency analysis of hypo/hypermethylated sites mentioned above, closer analysis of the top 500 most significantly differentially modified CpG sites across all conditions, identified two epigenetically modified clusters of interest (named Cluster A&B). Cluster B supported the frequency analysis above and demonstrated hypomethylation after load induced hypertrophy that was then maintained following unloading where muscle returned to baseline levels and this hypomethylation was then also maintained after reload induced hypertrophy. This maintenance of hypomethylation during unloading, suggested that the muscle ‘remembered’ the epigenetic modifications that occurred after an earlier period of load induced muscle hypertrophy. As reduced DNA methylation of genes generally leads to enhanced gene expression due to the removal of methylation allowing improved access of the transcriptional machinery and RNA polymerase that enable transcription, and also creating permissive euchromatin^19,28–30^, this would be suggestive that the earlier period of hypertrophy leads to increased gene expression of this cluster of genes that is then retained during unloading to enable enhanced muscle growth in the later reloading period. To confirm this, in a separate analysis we identified the top 100 most significantly differentially modified CpG sites across all conditions and cross referenced these with the most frequently occurring CpG modifications in all pairwise comparisons of experimental conditions. From this we identified 48 genes that were frequently occurring in all pairwise comparisons and examined gene expression by rt-qRT-PCR. Interestingly, we identified two clusters of genes with distinct temporal expression after loading, unloading and reloading. One of the clusters included AXIN1, GRIK2, CAMK4, TRAF1. Importantly, the majority of these genes demonstrated a mirror/inverse relationship with DNA methylation of the CpG sites within the same genes. Where DNA methylation reduced after loading and remained low into unloading and reloading, gene expression accumulated, demonstrating the highest expression after reloading where the largest increase in lean mass was also demonstrated. Overall, this suggested that these genes were hypomethylated and switched on after the earlier period of load induced hypertrophy, maintained during unloading due to methylation of these genes remaining low, and then upon exposure to a later period of reload induced hypertrophy, these genes were switched on to an even greater extent. Overall, this demonstrates that the methylation and collective responsiveness of these genes are important epigenetic regulators of skeletal muscle memory.

Interestingly, AXIN1 is a component of the beta-catenin destruction complex, where in skeletal muscle cells AXIN1 has been shown to inhibit WNT/β-catenin signalling and enable differentiation^31^, where treatment with the canonical WNT ligand suppresses differentiation^32^. Other studies suggested that AXIN2 not AXIN1 is increased after differentiation, however confirmed that the absence of AXIN1 reduced proliferation and myotube formation^32^. Therefore, together with the present data perhaps suggest an important epigenetic regulation of AXIN1 involved in human skeletal muscle memory and hypertrophy at the tissue level, perhaps due to inhibition of WNT/β-catenin signaling. GRIK2 (glutamate ionotropic receptor kainate type subunit 2, a.k.a. GluK2) belongs to the kainate family of glutamate receptors, which are composed of four subunits and function as ligand-activated ion channels^33^. Although reportedly expressed in skeletal muscle, its role in muscle growth or cellular function has not been determined. CAMK4 is calcium/calmodulin-dependent protein kinase, that via phosphorylation, triggers the CaMKK-CaMK4 signaling cascade and activates several transcription factors, such as MEF2^34^. MEF2 has been previously associated with a switch to slow fibre types after exercise^35^ and is hypomethylated after 6 months aerobic exercise^36^. While resistance exercise has been show to preferentially increase the size of type II faster fibres, chronic innervation even at higher loads can lead to an overall slowing in phenotype [reviewed in ref.^37^) and therefore this epigenetically regulated gene, although not usally studied during hypertrophy maybe important in fibre type changes in the present study. However, it is unknown how DNA methylation affects the protein levels of CAMK4, and with its role in phosphorylation, would be important to ascertain in the future. Furthermore, fibre type properties were not analyzed in the present study and therefore require further investigation. TRAF1 is the TNF receptor-associated factor 1 and together with TRAF2 form the heterodimeric complex required for TNF-α activation of MAPKs, JNK and NFκB^38^. In skeletal muscle, acute TNF exposure activates proliferation via activation of MAPKs such as ERK and P38 MAPK^39–41^. Therefore, acutely elevated systemic TNF-α following damaging exercise such as resistance exercise correlates positively with satellite cell activation *in-vivo* after damaging exercise^42,43^, yet chronic administration *in-vitro* inhibits differentiation, promotes myotube atrophy^40,44^ and muscle wasting *in-vivo*^44^. Indeed, exposure to early life TNF-α during an early proliferative age in mouse C2C12s results in maintenance of hypermethylation in the myoD promoter after 30 divisions and an increased susceptibility to reduced differentiation and myotube atrophy when muscle cells encounter TNF-α in later proliferative life^11^. Suggesting a role for DNA methylation in retention of memory following earlier periods of high inflammation. Because resistance exercise evokes increases in TNF-α in the systemic circulation and has been shown increase locally in muscle at the protein level (discussed above), these data collectively suggest an interesting epigenetic role for TNF and TRAF1 in the epigenetic memory of earlier load induced muscle hypertrophy.

### Identification of novel genes with the largest hypomethylation during reloading that are associated with enhanced gene expression

The second DNA methylation cluster determined in the top 500 differentially modified CpG sites across all conditions, identified a cluster of genes (named Cluster A) that was methylated at baseline and also became hypomethylated after loading (similar to Cluster B above), then, upon unloading, genes reverted back to a methylated state, and after reloading switched back to hypomethylated. Therefore, while not demonstrating an epigenetic memory per se, if hypomethylation was further enhanced and was associated with enhanced gene expression in reloading versus loading would also support an epigenetic memory. Further gene expression analysis identified a cluster of genes that demonstrated a mirror/inverse temporal pattern of gene expression versus their DNA methylation pattern. These genes included RPL35a, C12orf50, BICC1, ZFP2, UBR5, HEG1, PLA2G16, SETD3 and ODF2, that demonstrated hypomethylation of DNA after load induced growth and an increase in gene expression. Subsequently, then both DNA methylation and gene expression returned back to baseline levels (in opposite directions) and after reload induced muscle growth DNA was hypomethylated again with an associated increase in gene expression. Importantly, during reloading, gene expression was further enhanced versus loading, suggesting that an earlier period of load induced growth was enough to produce enhanced gene expression when reload induced muscle growth was encountered later, again suggesting a skeletal muscle memory at both the epigenetic and resultant transcript level. Statistical analysis identified the genes RPL35a, UBR5, SETD3 and PLA2G16 as having significantly enhanced expression upon reloading. Importantly, these four genes, plus HEG1, displayed significant correlations between their gene expression and the percentage change in lean mass, suggesting for the first time, a role for these four genes in regulating adult human load induced skeletal muscle growth. Interestingly, SET Domain Containing 3 (SETD3) is a H3K4/H3K36 methyltransferase, is abundant in skeletal muscle, and has been shown to be recruited to the myogenin promoter, with MyoD, to promote its expression^45^. Furthermore, overexpression of SETD3 in C2C12 murine myoblasts, evokes increases in myogenin, muscle creatine kinase, and Myf6 (or MRF4) gene expression. Inhibition via shRNA in a myoblasts also impairs muscle cell differentiation^45^, suggesting a role for SETD3 in regulating skeletal muscle regeneration. However, less is known regarding the role of PLA2G16 in skeletal muscle. PLA2G16 is a member of the superfamily of phospholipase A enzymes, whose predominant localization is in adipose tissue. PLA2G16 is known to regulate adipocyte lipolysis in an autocrine/paracrine manner, via interactions with prostaglandin and EP3 in a G-protein-mediated pathway^46^. Indeed, ablation of PLA2G16 (referred to as Adpla), prevents obesity during periods high fat feeding in mouse models, indicated via significantly less adipose tissue and triglyceride content, compared to relevant controls^46^. However, to date no known research has elucidated the role of PLA2G16 in skeletal muscle and therefore, this requires future experimentation. Finally, HEG homology 1 (HEG1), initially reported as the *heart of glass* gene, is recognised for its role in regulating the zebrafish heart growth. HEG1 is a transmembrane receptor that has been reported to be fundamental in the development of both the heart and blood vessels^47^. However, a recent study reported a distinct role for HEG1 in regulating malignant cell growth^48^. Tsuji, *et al*.^48^ and colleagues reported that gene silencing of HEG1 in human MPM cell line, a cell linage that develop mesothelioma tumours, significantly reduced the survival and proliferation of mesothelioma cells, suggesting a role for HEG1 in regulating cellular growth. However, no known research has examined the role of HEG1 in regulating adult skeletal muscle growth.

In the present study UBR5 displayed the most distinctive inverse relationship between DNA hypomethylation and increased gene expression following loading and reloading. With the largest increase in hypomethylation and gene expression after reloading where the largest increase in lean mass was observed. UBR5 is a highly conserved homologue of the drosophila tumour suppressor hyperplastic discs (HYD), and in the mammalian genome refers to a protein that is a member of the HECT-domain E3 ubiquitin-ligase family^49^. E3 ubiquitin ligases play an integral role in the ubiquitin - proteasome pathway, providing the majority of substrate recognition for the attachment of ubiquitin molecules onto targeted proteins, preferentially modifying them for targeted autophagy/breakdown^50^. Indeed, extensive work has identified a distinct role of a number of E3 ubiquitin ligases such as MuRF1, MAFbx and MUSA1 in muscle atrophy^51,52^. Furthermore, we have recently demonstrated that reduced DNA methylation and increased gene expression of MuRF1 and MAFbx are associated with disuse atrophy in rats following nerve silencing of the hind limbs via tetrodotoxin exposure^17^. A process that is reversed upon a return to habitual physical activity and a partial recovery of skeletal muscle mass^17^, suggesting a role for DNA methylation in regulating the transcript behavior of a number of ubiquitin ligases during periods of skeletal muscle atrophy and recovery. However, there have been no studies that the authors are aware of, exmaining the role of UBR5 in skeletal muscle atrophy or growth. Given the role of ubiquitin ligases in skeletal muscle, counterintuitively, we report that the expression of the E3 ubiquitin ligase, UBR5, is increased during earlier periods of skeletal muscle hypertrophy and are even further enhanced in later reload induced muscle growth. We further report that the methylation profile of this E3 ubiquitin ligase portrays an inversed relationship with gene expression, supporting a role for DNA epigenetic modifications in regulating its expression, as previously suggested^17^. However, in support of its role in positively impacting on muscle, UBR5 has also been shown to promote smooth muscle differentiation through its ability to stabilize myocardin proteins^53^. While myocardin is only expressed in smooth and cardiac muscle, it is considered the master regulator of smooth muscle gene expression^54^ and a known transcription factor that upregulates smooth muscle myosin heavy chains (MYHCs), actin and desmin. It therefore possesses a similar role to the myogenic regulatory factors during early differentiation (Mrf5 and MyoD), during fusion (myogenin) and during myotube hypertrophy (adult MYHC’s). Interestingly, it has previously been observed that myocardin-related transcription factors (MRTF) interact with the myogenic regulatory factor, MyoD, to activate skeletal muscle specific gene expression^55^, suggesting a potential cross-talk between muscle specific regulatory factors, enabling skeletal muscle adaptations^55,56^. Therefore, UBR5′s expression throughout the time course of skeletal muscle cell differentiation, its role in myotube hypertrophy are required *in-vitro* as well as mammalian overexpression and knock-out of UBR5 to confirm its importance *in-vivo*. Further work is needed to characterize UBR5, as well as other HECT-domain E3 ubiquitin ligase protein members identified in this work via pathway analysis of the ubiquitin mediated proteolysis pathway, in the development of muscle growth to better understand its role in facilitating skeletal muscle hypertrophy.

### A single bout of acute resistance exercise evokes hypomethylation of genes that have enhanced gene expression in later reload induced hypertrophy: Novel acutely exercise sensitive DNA methylation biomarkers

Finally, we identified genes BICC1, STAG1, GRIK2 and TRAF1 were hypomethylated after a single bout of acute resistance exercise that were maintained as hypomethylated during loading (as identified above) and reloading and demonstrated an enhanced gene expression after later reloading. Previous studies have suggested that acute aerobic exercise hypomethylates important genes in metabolic adaptation and mitochondrial biogenesis such as PGC-1α, mitochondrial transcription factor A (TFAM) and pyruvate dehydrogenase lipoamide kinase isozyme 4 (PDK4) post exercise, and reduces PPAR-δ methylation (hypomethylates) 3 hours post exercise^16^, with corresponding increases in gene expression (3 hrs post exercise for PGC-1α, PDK4 and PPAR-δ, immediately post for TFAM)^16^. Interestingly, hypermethylation of PGC 1α and reduced gene expression, observed in skeletal muscle of the offspring of obese murine mothers, was reversed (hypomethylated) by exercise in the mothers^4^. These data support the role for aerobic exercise in hypomethylating candidate genes. We also identify in the present study that hypomethylation (10,284 CpG sites) is favoured over hypermethylation (7,600 CpG sites) across the genome 30 minutes post an acute bout of resistance exercise, yet without changes in gene expression at this time point. Interestingly, however, hypomethylation of BICC1, STAG1, GRIK2 and TRAF1 after acute RE that was maintained after 7 weeks loading and reloading induced hypertrophy, resulted in significantly enhanced gene expression 22 weeks later. This suggested that DNA methylation of these genes after a single bout of resistance exercise were more sensitive biomarkers than their acutely corresponding gene expression for later load induced hypertrophy. BICC1 is an RNA binding protein that has an undermined role in adult skeletal muscle. It has been identified as differentially expressed during prenatal muscle development between two different pig breads^57^. RNA binding proteins in general are important in post transcriptional modifications, suggesting that perhaps reduced DNA methylation and increased gene expression may indicate an increase in post-transcriptional modification after reloading, however this requires further investigation to confirm. STAG1 (Cohesin subunit SA-1) is fundamental in cell division and part of the cohesin complex, which is required for the cohesion of sister chromatids after DNA replication^58^. However, to the authors knowledge there is no specific role for STAG1 identified in adult skeletal muscle hypertrophy. GRIK2 and TRAF2 were also identified as being hypomethylated after loading and reloading together with enhanced gene expression. As suggested above, GRIK2′s role in skeletal muscle is not well defined. However, TRAF1 has been widely implicated in skeletal muscle cell proliferation and differentiation, as discussed above, and hypomethylation of TRAF1 appears to be both sensitive to acute RE, as well as maintained following repeated loading and reloading induced hypertrophy that resulted in the largest increase in gene expression after reloading, 22 weeks after being detected as hypomethylated after acute RE. Overall, suggesting an important role for TRAF2 in skeletal muscles epigenetic memory of hypertrophy.

## Conclusion

We identify that human skeletal muscle possesses an epigenetic memory of earlier acute and chronic anabolic stimuli when encountering later muscle hypertrophy.

## Electronic supplementary material

Supplementary Figures 1,2,3,4 (SM1)

Supplementary File 1 (SM2)

Supplementary File 2 (SM3)

Supplementary File 3 (SM4)

Supplementary File 4 (SM5)

Supplementary File 5 (SM6)

Supplementary File 6 (SM7)

Supplementary File 7 (SM8)
