# Supplementary material for: Human Skeletal Muscle Possesses an Epigenetic Memory of Hypertrophy
Source: Sci Rep. 2018 Jan 30;8:1898. doi: 10.1038/s41598-018-20287-3 (PMC5789890; doi:10.1038/s41598-018-20287-3)
Supplement: Supplementary file 1 — Supplementary Figures 1,2,3,4 (SM1) [file 41598_2018_20287_MOESM1_ESM.pdf]

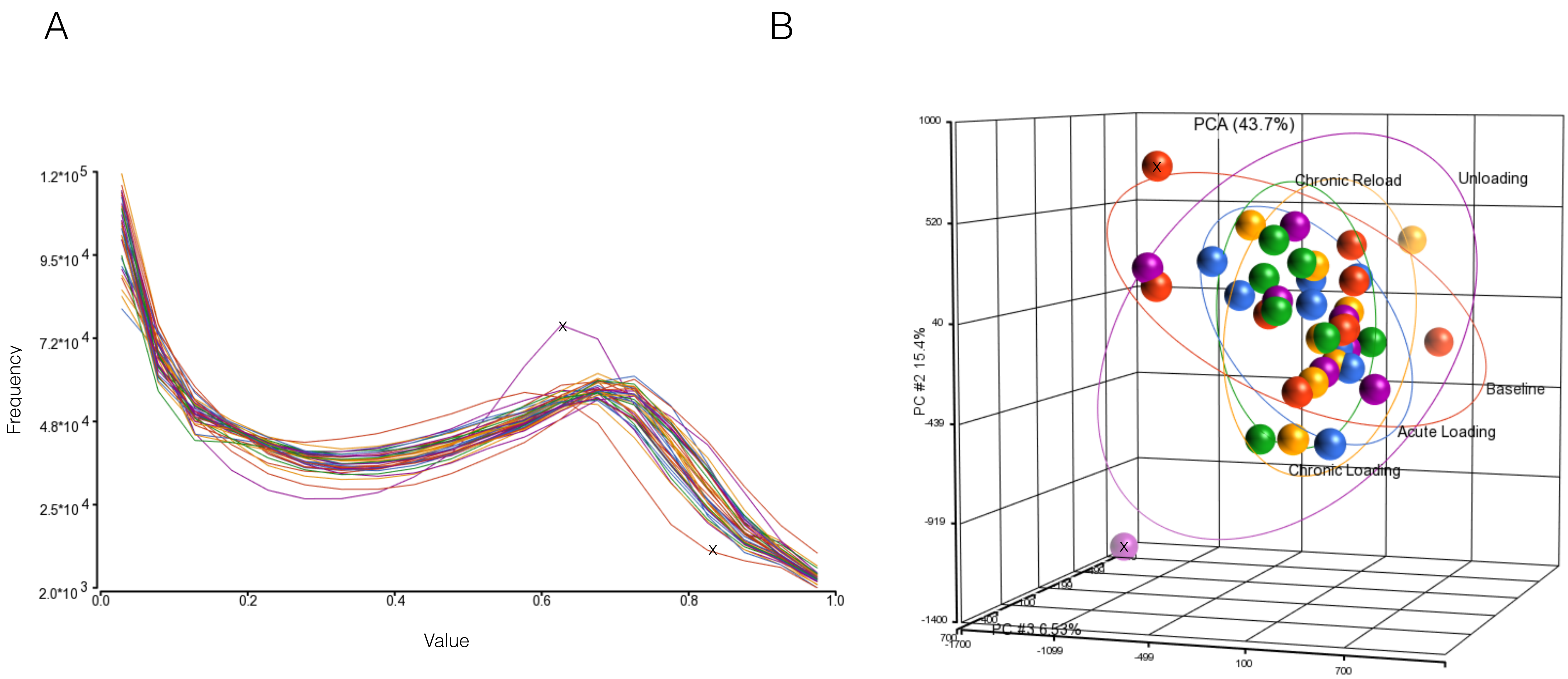

Supplementary Figure 1. Initial quality control analysis of human samples utilised for down-stream methylome wide bead arrays. **1a.** Histogram plot representing the distribution of intensity for each probe allowing for detection of aberrant samples. **1b.** Figure displaying the average methylation profile across all detected CpG loci allowing for analysis of variability using three-dimensional principal component analysis (PCA), where each sample is represented by a dot and ellipses for each condition. Two samples are identified as being outliers (X) from both quality control procedures and were subsequently removed from down-stream analysis.

Title: Human Skeletal Muscle Possesses an Epigenetic Memory of Hypertrophy

Authors: Robert A. Seaborne, Juliette Strauss, Matthew Cocks, Sam Shepherd, Thomas D. O'Brien, Ken A. van Someren, Phillip G. Bell, Christopher Murgatroyd, James, P. Morton, Claire E. Stewart, Adam P. Sharples

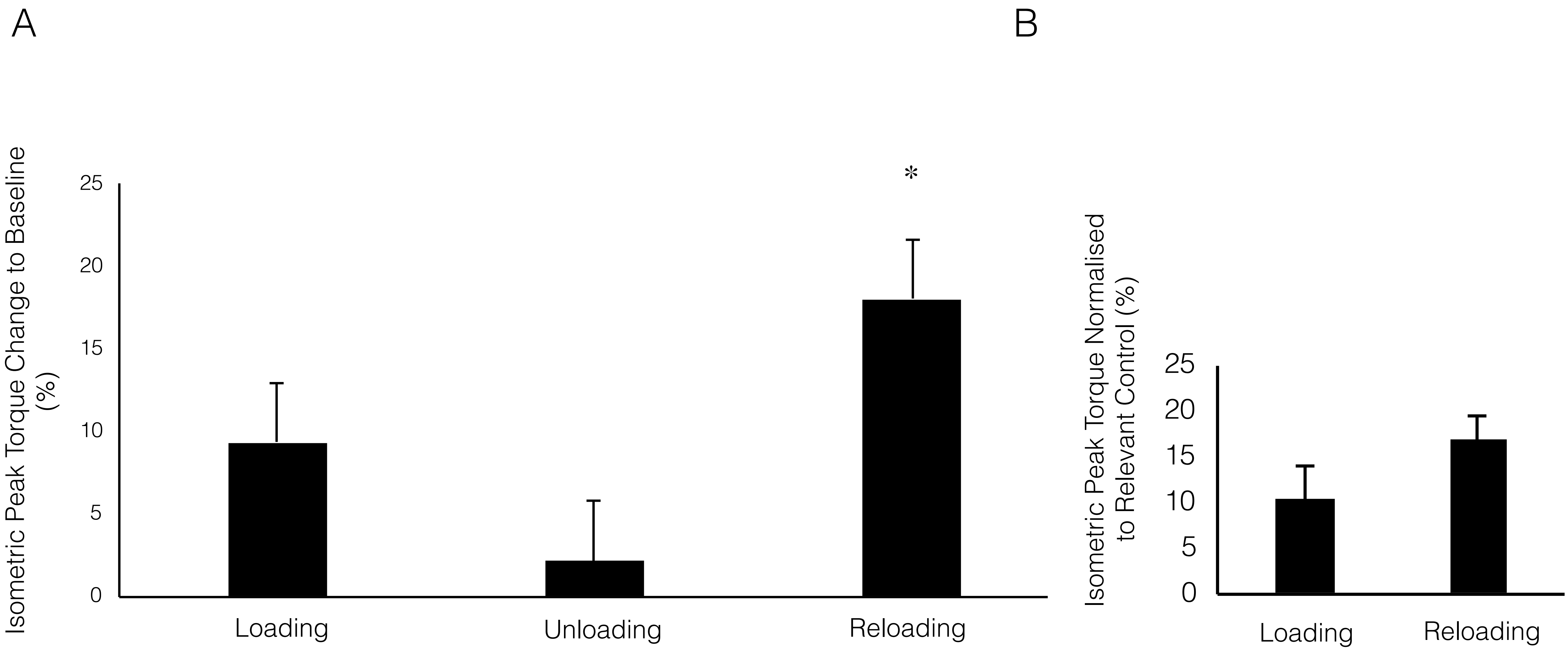

Supplementary Figure 2. Isometric quadriceps peak torque in human participants (n=7) after a period of 7 weeks' resistance exercise (loading), exercise cessation (unloading) and a subsequent second period of 7 weeks' resistance exercise (reloading) (Sup Fig 1a.). Significant change compared to baseline represented by *. **1b**. Isometric peak torque change when loading is normalised to baseline, and reloading normalised to unloading to account for any residual strength from the loading period. Pairwise t-test of significance indicated by *. All data presented as mean ± SEM (n=7).

Title: Human Skeletal Muscle Possesses an Epigenetic Memory of Hypertrophy

Authors: Robert A. Seaborne, Juliette Strauss, Matthew Cocks, Sam Shepherd, Thomas D. O'Brien, Ken A. van Someren, Phillip G. Bell, Christopher Murgatroyd, James, P. Morton, Claire E. Stewart, Adam P. Sharples

A

Loading Condition - PI3K/AKT Signalling Pathway

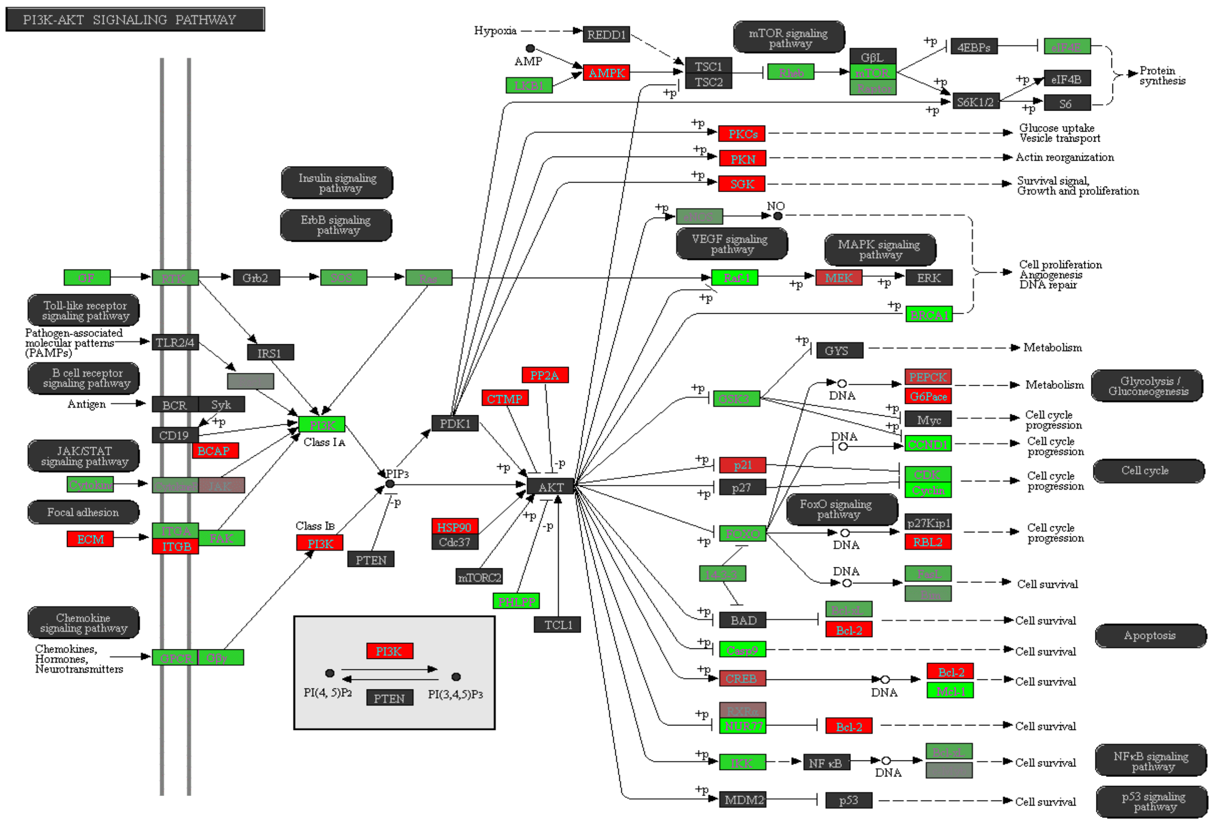

B

Unloading Condition - PI3K/AKT Signalling Pathway

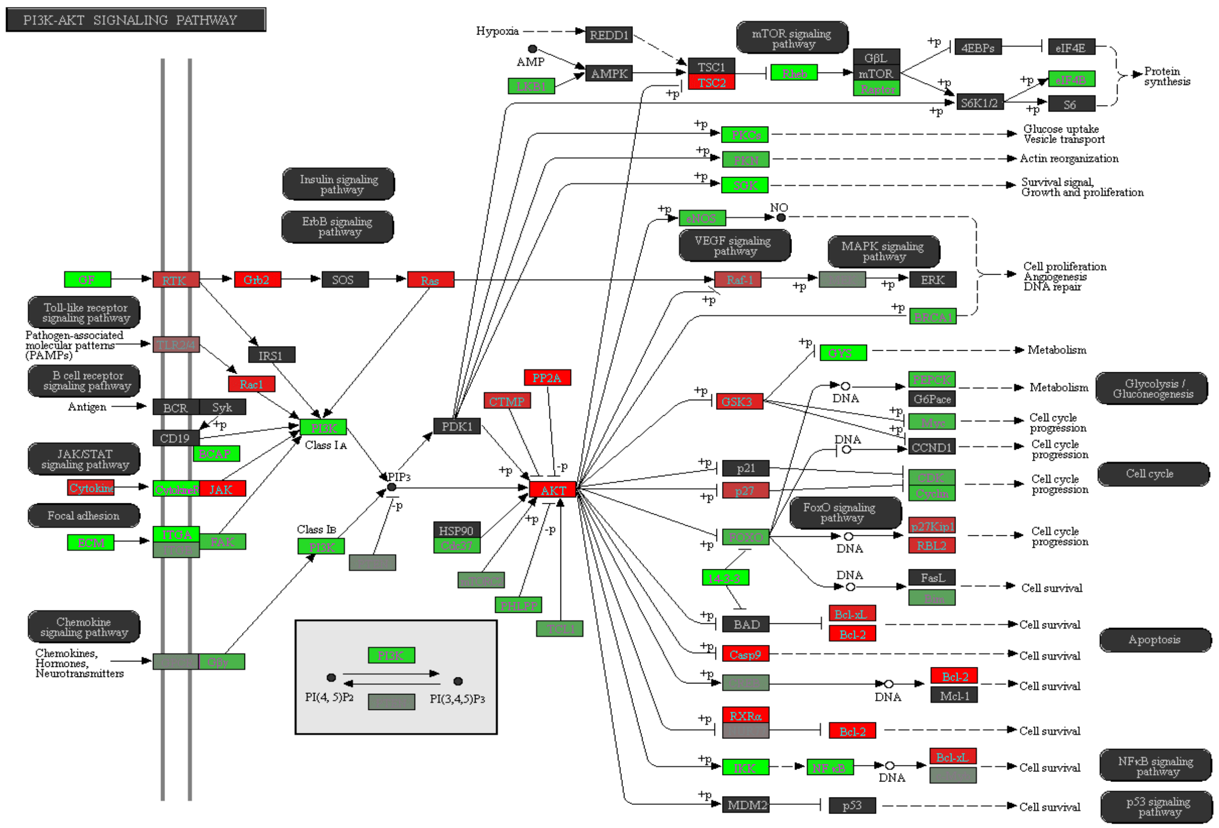

Supplementary Figure 3. Representation of the DNA methylation modifications that occurred within the PI3K/AKT signalling pathway following 7 weeks of loading (S**upp. figure 3a**) and unloading (S**upp. figure 3b**) in human subjects. Signalling analysis performed on statistically differentially regulated CpG sites compared to baseline, with green indicating a hypomethylated fold change and red indicating a hypermethylated change, with strength of colour representing the intensity of fold change [23-25].

Title: Human Skeletal Muscle Possesses an Epigenetic Memory of Hypertrophy

Authors: Robert A. Seaborne, Juliette Strauss, Matthew Cocks, Sam Shepherd, Thomas D. O'Brien, Ken A. van Someren, Phillip G. Bell, Christopher Murgatroyd, James, P. Morton, Claire E. Stewart, Adam P. Sharples

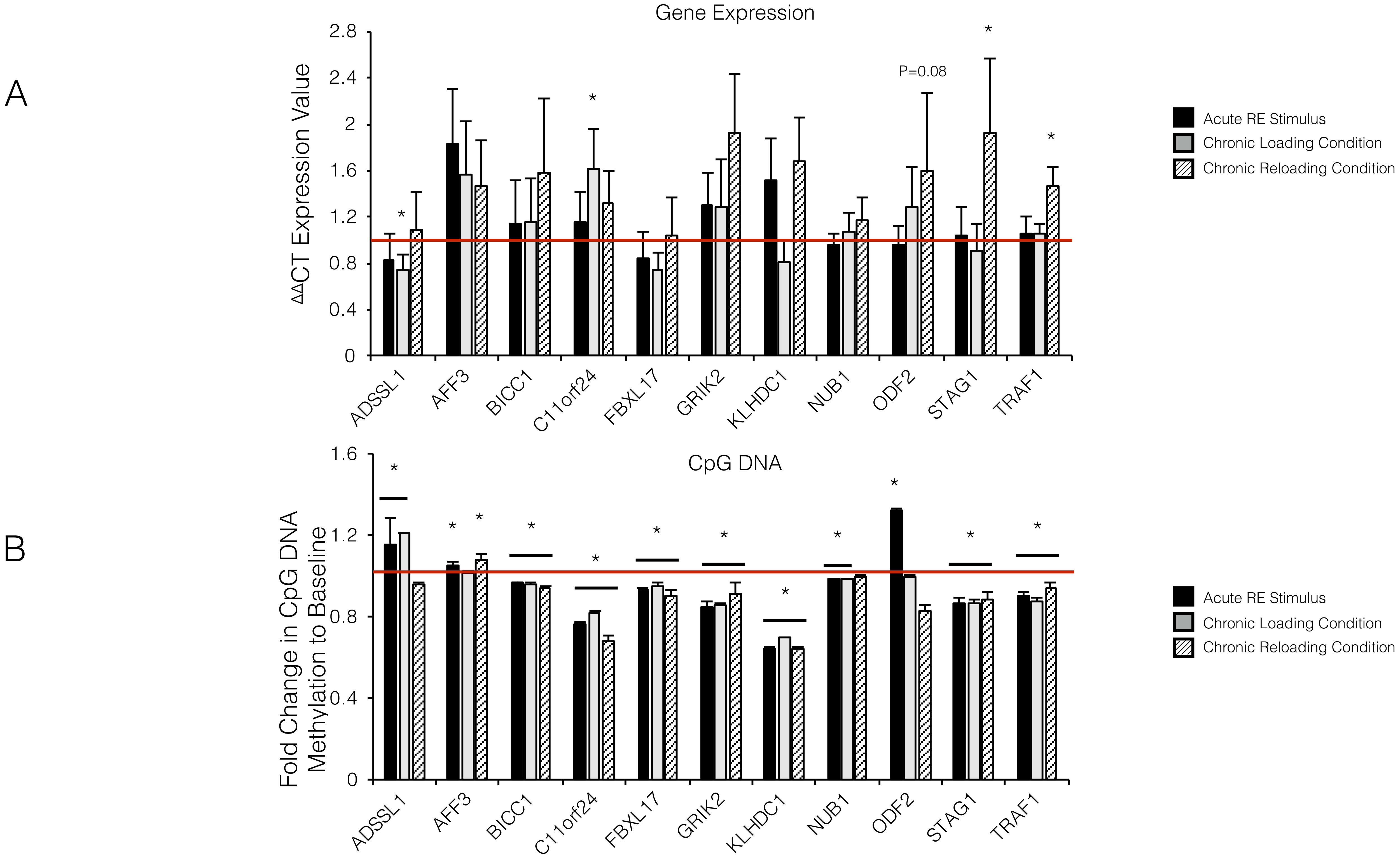

Supplementary Figure 4. Relative fold changes in; **a,** gene expression, and, **b,** CpG DNA methylation in all 18 gene transcripts identified from comparison of methylome data from acute RE vs. 7 weeks loading, unloading, reloading analysis. Data represented as means ± SEM, with significance compared to baseline (red line) indicated by *.

Title: Human Skeletal Muscle Possesses an Epigenetic Memory of Hypertrophy

Authors: Robert A. Seaborne, Juliette Strauss, Matthew Cocks, Sam Shepherd, Thomas D. O'Brien, Ken A. van Someren, Phillip G. Bell, Christopher Murgatroyd, James, P. Morton, Claire E. Stewart, Adam P. Sharples
