# Supplementary material for: Human Skeletal Muscle Possesses an Epigenetic Memory of Hypertrophy
Source: Sci Rep. 2018 Jan 30;8:1898. doi: 10.1038/s41598-018-20287-3 (PMC5789890; doi:10.1038/s41598-018-20287-3)
Supplement: Supplementary file 2 — Supplementary File 1 (SM2) [file 41598_2018_20287_MOESM2_ESM.doc]

**Human Skeletal Muscle Possesses an Epigenetic Memory of Hypertrophy**

Robert A. Seaborne^1, 2^, Juliette Strauss^2^, Matthew Cocks^2^, Sam Shepherd^2^, Thomas D. O’Brien^2^, Ken A. van Someren^3^, Phillip G. Bell ^3^, Christopher Murgatroyd ^4^, James, P. Morton^2^, Claire E. Stewart^2^, Adam P. Sharples^1, 2 #^

^1^ Institute for Science and Technology in Medicine (ISTM), School of Medicine, Keele University, Staffordshire, United Kingdom.

^2^ Research Institute for Sport and Exercise Sciences, Liverpool John Moores University, Liverpool, United Kingdom.

^3^ Department of Sport, Exercise and Rehabilitation, Northumbria University, Newcastle upon Tyne, United Kingdom.

^4^ School of Healthcare Science, Manchester Metropolitan University, Manchester, United Kingdom

# Corresponding/Senior Author

Address for correspondence:

Dr. Adam P. Sharples

Institute for Science and Technology in Medicine,

Guy Hilton Research Centre, Thornburrow Drive, Hartshill,

University of Keele,

Stoke-on-Trent,

ST4 7QB,

United Kingdom

Email: [a.p.sharples@googlemail.com](mailto:a.p.sharples@googlemail.com)

**Supplementary File 1: Quadriceps Muscle Strength Measurements**

To assess quadriceps muscle strength, *in-vivo* isometric knee extension maximal voluntary contractions (MVC) were performed using an isokinetic dynamometer (IKD; Biodex, New York, USA) to measure peak joint torque. Data presented as percentage increase to baseline (%) using absolute values (Nm), unless otherwise stated. The participants were seated on the dynamometer chair with the hip flexed to 90°, the lateral femoral condyle of the right leg aligned with the dynamometer axis of rotation during contraction and the tibia strapped to the dynamometer lever arm proximal to the malleoli. Inextensible straps harnessed participants into the chair at the hip, distal end of the thigh and the chest to minimize compensatory movements. Following a self-selected warm-up of submaximal and maximal contractions, participants performed two or three isometric MVCs at the ‘optimum’ knee angle for torque production. This angle was identified as that at which peak torque was recorded during a prior isokinetic knee extension at a constant speed of 30°/s (through a full range of motion). The optimum ranging between 70^o^ – 90^o^. A rest period of 90 – 120 s was allowed between efforts. Verbal and visual (real-time trace of torque motion) feedback as well as verbal encouragement were given throughout all MVC efforts. Joint torques and angles were recorded and filtered with a low-pass 500 Hz filter (AcKnowledge Version 4.4) to omit background noise. The greatest peak joint torque recorded from all isometric trials was taken forward for analysis. Data presented as percentage increase to baseline (%) using absolute values (Nm), unless otherwise stated.
